# Light-Mediated Desulfurization of Peptides in Multi-Well Format for Combinatorial Library Synthesis

**DOI:** 10.3390/molecules31173097

**Published:** 2026-09-03

**Authors:** Esther Arentoft Jacobsen, Julian Lava, Anders Thorseth, Dennis Dan Corell, Carsten Dam-Hansen, Charlotte Held Gotfredsen, Martin Kræmer, Poanna Tran, Katrine Qvortrup

**Affiliations:** 1 Gubra, 2970 Hørsholm, Denmark; esther.a.jacobsen@gmail.com (E.A.J.); mkr@gubra.dk (M.K.); 2 Department of Chemistry, Technical University of Denmark, 2800 Kongens Lyngby, Denmark; julava@kemi.dtu.dk (J.L.); chg@kemi.dtu.dk (C.H.G.); 3 Department of Electrical and Photonics Engineering, Technical University of Denmark, 2800 Kongens Lyngby, Denmark; andt@dtu.dk (A.T.); ddco@dtu.dk (D.D.C.); cadh@dtu.dk (C.D.-H.)

**Keywords:** photochemistry, cysteine desulfurization, peptide synthesis, high-throughput synthesis, native chemical ligation, macrocyclization

## Abstract

Native chemical ligation (NCL) combined with post-ligation desulfurization is a powerful strategy for the chemical joining of unprotected peptide segments to form larger polypeptides or to synthesize head-to-tail cyclized peptides. However, a simple, robust, and safe desulfurization protocol suitable for high-throughput synthesis workflows has yet to be developed. Here, we present a photochemical strategy in which desulfurization is achieved using 270 nm ultraviolet (UV) irradiation in the presence of tris(2-carboxyethyl)phosphine (TCEP). Optimization of reaction conditions resulted in reaction efficiency comparable with current chemical desulfurization methods. Desulfurization of a combinatorial peptide library confirmed that the photochemical desulfurization strategy is compatible with all canonical amino acids as well as thiol-protected cysteines. Implementation of the optimized conditions in a 96-well plate format using a custom-built light-emitting diode (LED) array confirmed that product formation is dependent on local light exposure. Increasing the distance between the plate and the light source enabled more uniform irradiation, giving homogenous product formation. Altogether, this study establishes UV-mediated desulfurization as a safe and scalable alternative for high-throughput peptide synthesis workflows.

## 1. Introduction

Peptides have attracted increasing interest as pharmaceuticals, and the parallel advancement in peptide synthesis have accelerated peptide drug discovery resulting in the approval of more than one hundred peptide-based drugs. Peptides often have higher target specificity than small molecules while being less immunogenic than biologics, making peptides attractive candidates in drug development [1,2,3]. To increase metabolic stability, cyclized peptides are now particularly seen as promising drug candidates [4,5,6,7,8].

Native chemical ligation (NCL) is a widely used method for head-to-tail cyclization of peptides. This strategy utilizes an N-terminal cysteine and a C-terminal thioester for ligation, resulting in a native peptide bond and a cysteine residue at the ligation site [9]. The C-terminal thioester can preferably be replaced by a surrogate group that is stable under the standard conditions used for 9-fluorenylmethyloxycarbonyl (Fmoc) solid-phase peptide synthesis (SPPS). This has been achieved by attaching the peptide to the resin through *N*-acylurea linkers or the use of Weinreb amide derivatives, or hydrazides [10,11,12,13]. NCL is performed under mild conditions (typically in aqueous solution near neutral pH) using unprotected peptides.

Desulfurization of peptides following NCL to convert cysteine at the ligation site to alanine enables native peptide sequences to be obtained following ligation procedures. Ligation using unnatural amino acids bearing a thiol in the beta- or gamma-position followed by desulfurization has facilitated the synthesis of “scar-free” cyclic peptides with a broader range of canonical amino acids at the ligation site, including aspartate, glutamate, glutamine, leucine, and valine [14,15,16,17,18].

Strategies for chemical desulfurization of peptides use radical initiators, such as VA-044 or boron-based reagents, in combination with water-soluble phosphine reagents such as tris(2-carboxyethyl)phosphine (TCEP) [19,20,21,22]. Notably, a superfast protocol using NaBEt_4_ was shown to reduce the reaction time from hours to seconds. However, due to its pyrophoric nature and acidic pH requirement, this reagent poses challenges for safe and efficient handling in high-throughput syntheses. Therefore, other strategies for desulfurization have been explored. Since desulfurization proceeds through sulfur radical intermediates, alternate strategies capable of generating these radicals under mild conditions are desirable.

Examples include mechanically triggered ultrasound-induced formation of thiyl radicals using an ultrasonic bath with sonosensitizers [23]. Electrochemistry has also emerged as a strategy for desulfurization of peptides, forming a thiyl radical cation via anodic oxidation using platinum electrodes [24].

Photochemical desulfurization protocols have also been developed using visible light from household light bulbs or light-emitting diodes (LEDs) in combination with a photoredox catalyst or radical initiator and a phosphine reagent, with irradiation times ranging from 3 to 16 h [25,26,27].

Strategies using ultraviolet (UV) light without photocatalysts, radical initiators, or photosensitizers have been demonstrated as well. Chisholm et al. reported an efficient flow-based method enabling desulfurization of peptides upon 254 nm irradiation from a 35 W photoreactor in the presence of TCEP. Irradiation with longer wavelength light (365 nm) also resulted in desulfurization, but with slower conversion [28]. Photochemical desulfurization using 365 nm light has been further explored by Venneti et al. In this work, irradiation at 365 nm gave full conversion within 30 min, employing triphenylphosphine (PPh_3_) as the phosphine reagent in tetrahydrofuran (THF) [29].

**Figure 1:**
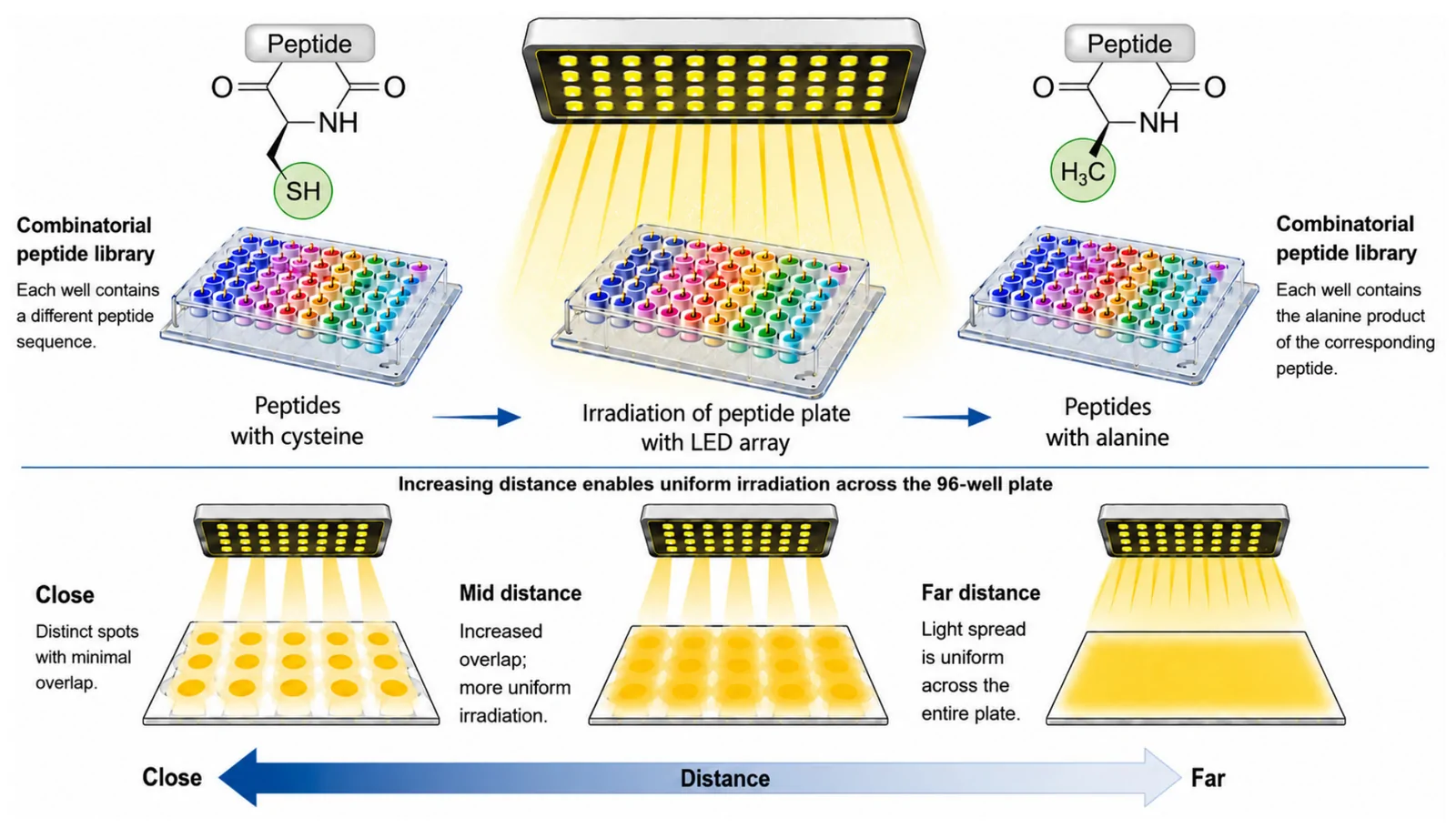
Schematic overview of the high-throughput photochemical desulfurization workflow. A combinatorial peptide library containing cysteine residues is irradiated using a UV LED array to generate the corresponding alanine-containing peptides. Increasing the distance between the LED array and the 96-well plate improves light overlap, resulting in more uniform irradiation across the plate.

As presented above, photochemistry shows great promise for the desulfurization of polypeptides and proteins. Photochemical desulfurization not only expands the toolbox of peptide chemistry but provides a simple, safe desulfurization method. This study investigated the desulfurization of peptides using UV light and TCEP in aqueous solution with the aim of developing a streamlined process for high-throughput synthesis of peptides that are head-to-tail cyclized or ligated via NCL. The optimized protocol was assessed in comparison with a standard NaBEt_4_-based protocol. Finally, this work explored the potential of a UV LED array for the desulfurization of peptides in plate format (Figure 1).

## 2. Results and Discussion

### 2.1. Optimization of Reaction Conditions

The efficiency of the light-mediated peptide desulfurization depends on several experimental factors including wavelength of irradiation, buffer composition, and pH. These variables were systematically optimized to establish the conditions providing rapid and efficient desulfurization. Optimization of reaction conditions was carried out using a linear peptide with the sequence LNDSACALVEE-NH_2_. The peptide was designed to contain a central cysteine residue as a surrogate for a peptide that has undergone inter- or intramolecular NCL. The peptide contained alanine residues in the positions adjacent to the reacting cysteine to avoid any potential interference with amino acids bearing bulky or reactive side chains in the initial optimization.

Two single LEDs with different wavelengths from Thorlabs were tested. One LED had a nominal wavelength of 265 nm and an optical output power of 55.7 mW when operated at maximum current (440 mA) [30] and a measured peak wavelength of 267.9 nm, while the other LED had a nominal wavelength of 275 nm and an optical output power of 80.0 mW when operated at maximum current (700 mA) [30] and a measured peak wavelength of 280.2 nm (Figure S1a,b).

For the initial experiments, 35.3 nmol batches of the model peptide were dissolved in 100 mM phosphate buffer (PB) at pH 7 (0.88 mM) and irradiated for 30 min. All samples were analyzed quantitatively by liquid chromatography mass spectrometry (LCMS) based on peak area from the UV trace acquired at 220 nm. Comparing irradiation with 265 nm and 275 nm, when both operated at maximum current, it was found that the shorter wavelength provided notably faster conversion (Table 1), even though spectral power distributions of the two LEDs showed that the 275 nm LED has a higher photon flux density (5.15 μmol/m^2^·s) compared to 265 nm LED (3.66 μmol/m^2^·s) (Figure S1c). Thus, the 265 nm LED was employed for the remaining experiments. Looking at TCEP concentrations, it was found that both 10 and 20 equiv. yielded nearly full conversion and therefore, all further desulfurization experiments used 10 equiv. TCEP unless stated otherwise.

Next, the effect of buffers containing acetonitrile (MeCN) was evaluated (Table 1). MeCN is widely used for peptide handling and purification to enhance dissolution of hydrophobic peptides [31,32] and as such, inclusion of MeCN would enable the protocol to be applied to peptide sequences with varying physicochemical properties. The efficiency of the reaction decreased noticeably with an increasing concentration of MeCN. Based on work by Venneti et al. [29], 10 equiv. *N*,*N*-diisopropylethylamine (DIPEA) were added in an attempt to increase the efficiency of the reaction via the deprotonation of TCEP in the MeCN-containing buffers. However, the addition of DIPEA to the buffer systems did not increase product formation markedly.

Because the irradiation setup was an open system, it was not possible to conduct experiments under fully anaerobic conditions. Nevertheless, the effect of bubbling nitrogen through the solvent prior to irradiation was investigated (Table 1). Samples in degassed aqueous buffer containing 5 eq. TCEP increased product formation from 78% to 86%. Similarly, degassed samples containing 25% MeCN and 50% MeCN with 10 eq. TCEP increased product formation from 44% to 68% and from 23% to 54%, respectively. This suggests that the higher solubility of oxygen in MeCN [33], leads to increased quenching of radical intermediates, reducing product formation in buffers containing MeCN.

Following evaluation of solvent composition, phosphate buffers with varying pH (6, 7, and 8) were tested (Table 1). To avoid full conversion within the irradiation period, allowing better comparison of the different conditions, the TCEP concentration was decreased from 10 to 5 equiv. It was found that decreasing the pH from 7 to 6 gave a notable decrease in product formation; however, pH 7 and 8 gave comparable product formation. Since thiyl radicals are formed more readily from thiolates than from protonated thiols [34,35], increasing the pH was expected to enhance product formation by increasing the concentration of the reactive thiolate species.

Given these results, higher pH values were investigated using a tris(hydroxymethyl)aminomethane (Tris) buffer with pH 7, 8, or 9. It was found that a higher pH led to an increase in product formation. Based on these results, we attempted to accelerate the reaction in a MeCN-containing buffer by increasing the pH. However, product formation did not vary between samples with varying pH, and the percentage of peptide converted to product after irradiation was only 8–9%.

Based on these optimization studies, irradiation at 265 nm in PB containing 10 equiv. TCEP was selected as the standard desulfurization protocol. Although increasing the pH accelerated the reaction, subsequent experiments were performed at pH 7 to maintain compatibility with the ligation conditions. NMR-based analysis of the model peptide **1** and the product **2** confirmed the reaction and product formation. A table of the complete ^1^H, ^13^C, and ^15^N resonance assignments (in DMSO-*d6*) and overview spectra can be found in the Supplementary Material, for the model peptide **1** (S31–37) and the product **2** (S38–44). The conversion of the Cys to an Ala is readily visible in the spectra, both displaying different chemical shifts for the Cys CH_2_ (δ_H_—2.76/2.68 ppm; δ_C_—26.0 ppm) and the Ala CH_3_ (δ_H_—1.24 ppm; δ_C_—18.0 ppm). A sequential connectivity along the peptide chain confirms the amino acid sequence.

### 2.2. Quantitative Analysis of Reaction Efficiency

Following optimization of reaction parameters, the efficiency of the light-mediated desulfurization reaction was compared between the linear model peptide and its cyclic analog. For this analysis, a lipophilic peptide was added as an internal standard, and peak areas of product and starting material were normalized to the standard. After 30 min irradiation, 71% of the linear peptide was desulfurized with 4% of the starting material remaining. Total peak area including byproducts was determined to be the same before and after the reaction, suggesting that the remaining 25% of the peptide was converted into unwanted byproducts. A similar trend was observed for the cyclic analog displaying 71% desulfurized product and 7% starting material. Thus, light-mediated desulfurization is suitable for head-to-tail cyclized peptides (Figure S10). The newly developed protocol was compared with the superfast desulfurization strategy presented by Sun et al. employing 20 equiv. NaBEt_4_ and 5 equiv. TCEP. Exposing the cyclic model peptide to both sets of conditions revealed that the reactions presented similar efficiencies with 64% conversion using the boron reagent, making the light-mediated desulfurization slightly more efficacious (Figure S11). It should be noted that the chemical desulfurization was complete within seconds after addition of NaBEt_4_, whereas the light-mediated desulfurization required 30 min of irradiation. Again, the total peak area of the chemical desulfurization reaction suggested that 36% of the peptide was converted into unwanted byproducts.

### 2.3. Sequence Scope and Functional Group Tolerance

**Figure 2:**
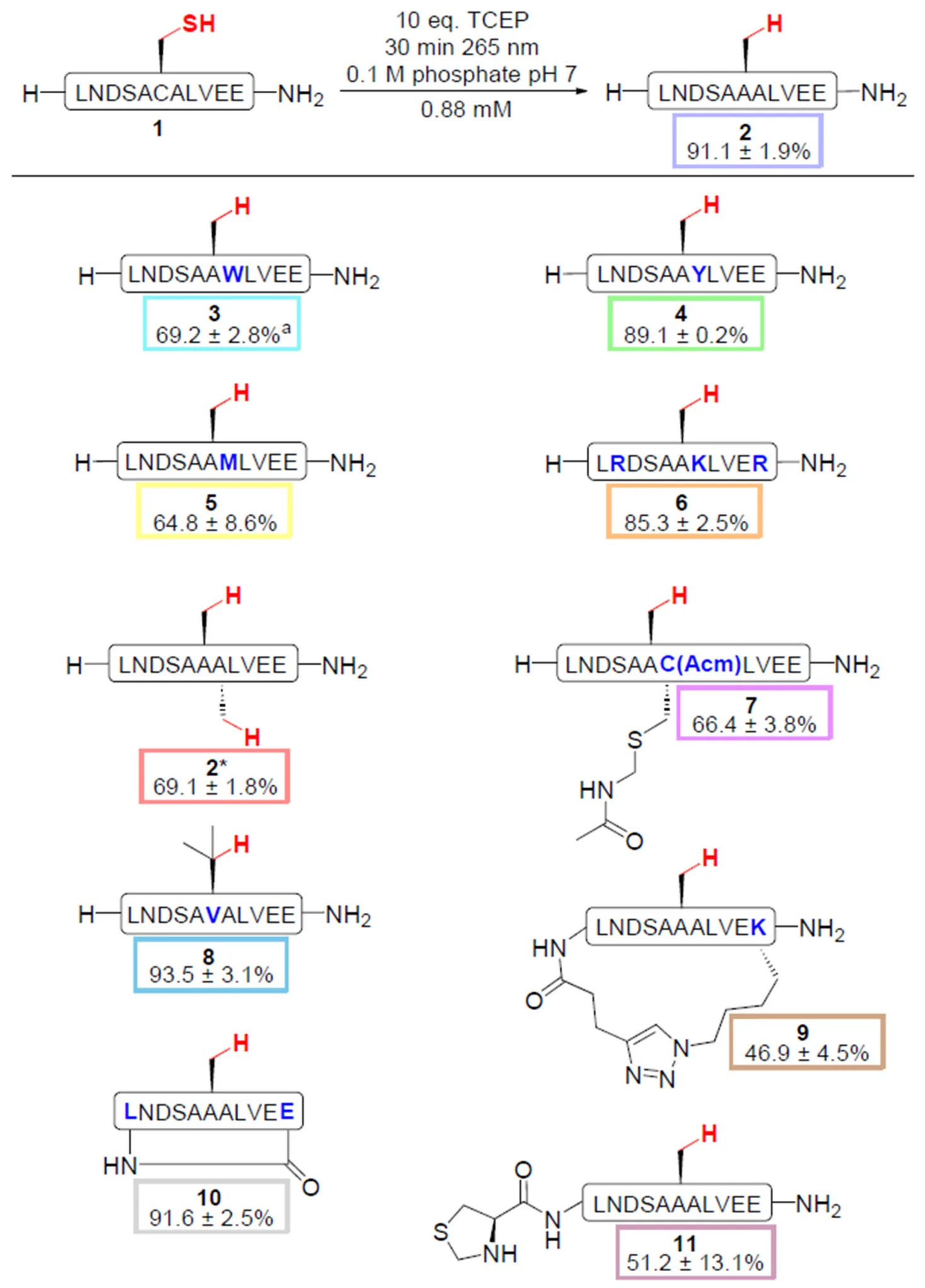
Conversion of various peptide substrates (35.3 nmol) following light-mediated desulfurization employing 10 eq. TCEP in 0.1 M PB after 30 min of irradiation. Side chains resulting from desulfurization are indicated in red. Residues differing from **2** are indicated in blue. ^a^ Peptide **3** was subjected to irradiation for 10 min. 2* indicates a peptide identical to **2** resulting from desulfurization of two Cys residues. The percentage of formation of 2* includes only fully desulfurized product. The percentage of formation of **11** includes all desulfurized product, including thiazolidine deprotection and subsequent double desulfurization. Values are shown as mean conversion percentage ± SD (Figure S12).

To evaluate the applicability of the light-mediated desulfurization protocol, a combinatorial library of eleven peptides was synthesized (Figure 2). The library included peptides containing aromatic (Trp, Tyr) or basic (Lys, Arg) amino acids, as well as sequences incorporating two cysteine residues, thioethers (Met, Cys-acetamidomethyl (Acm), thiazolidine (Thz)), or penicillamine, which results in a Val residue after desulfurization. In addition, a peptide bearing a 1,2,3-triazole ring was evaluated.

Notably, the efficiency of the reaction was found to vary depending on peptide sequences and functional group composition (Figure 2). Formation of **3** was found to proceed more rapidly than the formation of other substrates with complete consumption of starting material after 30 min of irradiation. However, irradiation also resulted in the formation of the oxidized product. Therefore, conversion percentages were determined after 10 min of irradiation to minimize byproduct formation, although a small amount of the oxidized product (3.2 ± 0.1%) was already observed at this time point. Peptides **4**, **6**, **8**, and **10** exhibited conversions exceeding 85%, whereas peptide **9** displayed notably lower conversion (<50%). During irradiation, peptide **11** formed a byproduct exhibiting a mass 44 Da lower than the desulfurized product, potentially arising from Thz deprotection and subsequent desulfurization. Rewardingly, it was found that thioethers such as methionine and Acm-protected cysteine (**5** and **7**) remained intact under the desulfurization conditions, and the simultaneous desulfurization of two cysteine residues (2*) was found to be feasible. These results collectively demonstrate that peptides incorporating diverse functional groups can be desulfurized using TCEP and 265 nm UV irradiation.

### 2.4. Light-Mediated Desulfurization in Plate Format

High-throughput approaches offer the potential to accelerate synthesis and evaluation of diverse peptide sequences in drug discovery. For the light-mediated desulfurization to be implemented in high-throughput workflows, the reaction must be compatible with a plate format.

**Figure 3:**
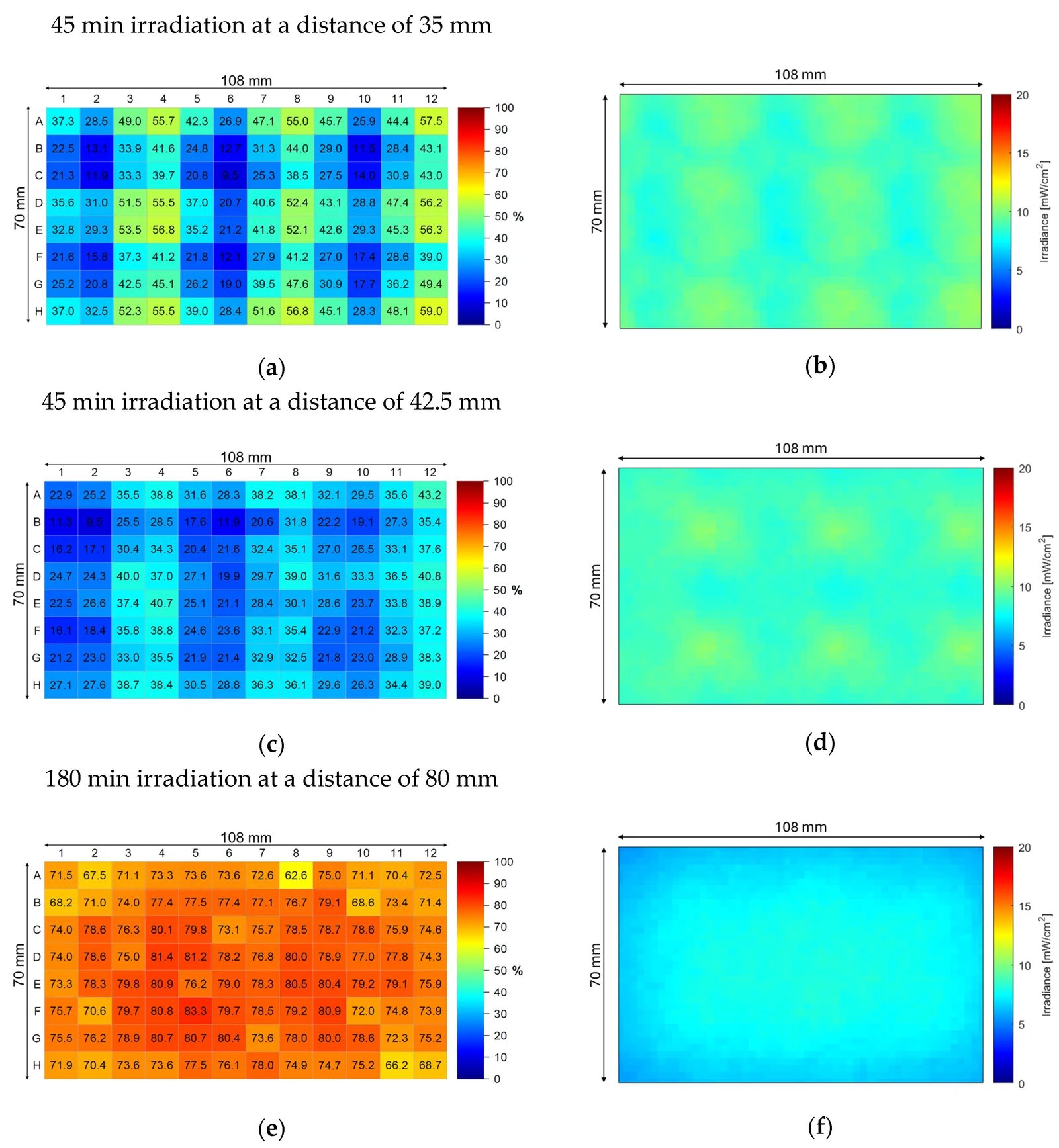
Heatmaps of percentage of product formation after irradiation (*n* = 1) (**left**) and calculated absolute irradiance distribution supplied by the first-generation UV lamp fitted within well plate alignment and dimensions (**right**). (**a**) Product after 45 min at a distance of 35 mm. (**b**) Irradiance distribution at 35 mm. (**c**) Product after 45 min at 42.5 mm. (**d**) Irradiance distribution at 42.5 mm. (**e**) Product after 180 min at 80 mm. (**f**) Irradiance distribution at 80 mm.

Thus, the reaction was investigated using 0.43 μmol batches of peptide **1** in 0.1 M PB with 10 equiv. TCEP at pH 7 in 96-well 0.5 mL deep well plates. Although initial single LED experiments with 265 nm showed higher conversion compared to 275 nm, the limited commercial availability of single chip 265 nm LEDs suitable for custom array fabrication necessitated the use of 270 nm emitters. A custom-built 3 × 4 LED array emitting at 270 nm (Figure S13) was used to irradiate the plate with an average irradiance of 8.98 mW/cm^2^ (peak irradiance of 10.65 mW/cm^2^) across the well plate area at a 35 mm distance. The plate was positioned underneath the LED array with a distance of 35 mm between the top of the plate and the LEDs. Irradiation for 45 min gave an average product yield of 35.7 ± 13.0% (Figure S14) across the plate. Heat maps of product formation and calculated irradiance revealed a strong dependence on local light exposure with a clear spatial correlation between irradiance and product formation (Figure 3a,b). Due to non-uniform product formation across the plate, the experiment was repeated at a greater distance between the plate and the LED array to achieve more uniform irradiation, although also reducing irradiance exposure.

Irradiation at 42.5 mm for 45 min resulted in 29.1 ± 7.5% product formation (Figure S15). Increasing the distance from the LED array thus enabled more uniform product formation but as expected, also decreased the overall rate of conversion (Figure 3c,d). Nevertheless, the heat map continued to exhibit localized regions of lower product formation reflecting the spatial arrangement of the LEDs.

Calculations of the irradiance at 80 mm from the first-generation UV lamp revealed that this configuration should improve uniform light exposure across the entire plate (Figures S16 and S17). However, increasing the distance substantially reduces the reaction rate, necessitating longer irradiation times to evaluate product formation consistency (Figure 3e,f). After 45 min of irradiation, samples from 10 randomly selected wells showed only 10.4 ± 4.7% product formation (Figure S18). Continued irradiation for an additional 135 min increased product formation to 75.9 ± 3.9% (Figure S19). Initially, the plate was not precisely centered underneath the array reflecting the asymmetric product formation across the plate. From this experiment onwards, the positioning of the 96-well plate was optimized by carefully centering it beneath the LED array.

UV transmission measurements of the polypropylene (PP) well plate (Figure S20) confirm near-zero light transmission below 300 nm, caused by the high UV absorbance of the PP material [36]. Consequently, the well walls act as a barrier to angled light, restricting sample irradiation to direct top-down exposure and contributing to localized shading effects within the wells. For future high-throughput production, utilizing UV-transparent material (such as quartz or UV-compatible cyclic olefins) would allow higher angled light penetration, significantly increase overall irradiance and improve reaction productivity.

These results demonstrate a direct correlation between irradiation and product formation, establishing irradiance as a key parameter governing reaction efficiency. While increasing the distance between the LED array and the plate improved conversion uniformity, the resulting decrease in irradiance led to slower reaction kinetics. With this insight, a new LED array was designed to incorporate more LEDs to enable high conversion within shorter irradiation periods while maintaining homogeneous irradiation.

**Figure 4:**
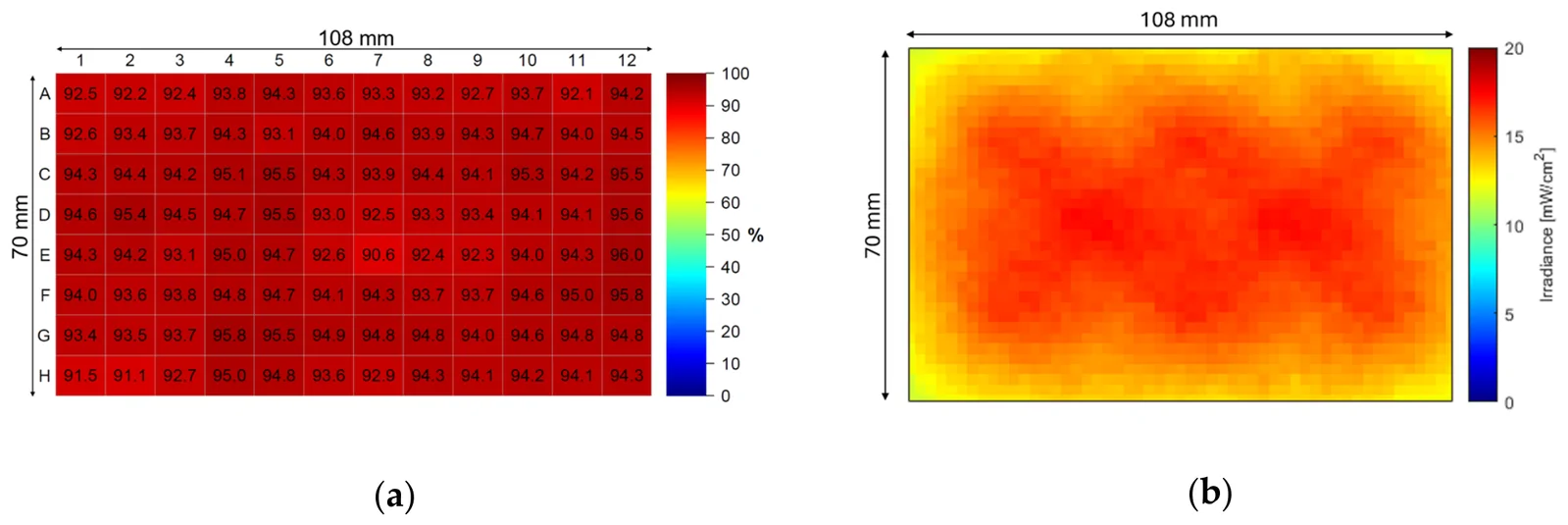
(**a**) Heatmap of percentage of product formation after 130 min at a 50 mm distance from the LED array (*n* = 1). (**b**) Calculated absolute irradiance supplied by the second-generation UV lamp at 50 mm.

Using a second-generation lamp comprising a 3 × 4 plus 2 × 3 LED array (Figure S13), it was possible to irradiate plates at a higher irradiance, resulting in faster conversion (Figure 4). Heatmaps of irradiance at varying distances from the new lamp system revealed that performing the next experiment at 50 mm would provide a good balance between reaction efficiency and homogeneity (Figures S21 and S22). Subjecting a full plate to 15.01 mW/cm^2^ (peak irradiance of 17.37 mW/cm^2^) at a distance of 50 mm for 130 min led to 94.0 ± 1.0% conversion. The uniform product formation observed after 130 min was also evident at earlier irradiation times (100 and 115 min) despite incomplete conversion (Figures S23–S26). It should be noted that faster conversion could be achieved by placing the plate closer to the LED array. However, this also resulted in more heterogenous product formation across the plate (Figures S27–S30). To ensure that prolonged irradiation did not cause thermal degradation of the peptides, the temperature was monitored over a 130 min period for four water samples irradiated at a 35 mm distance (Figure S31). During irradiation, the internal temperature for each vial remained below 26 °C, demonstrating that the heating caused by the lamp was negligible.

**Figure 5:**
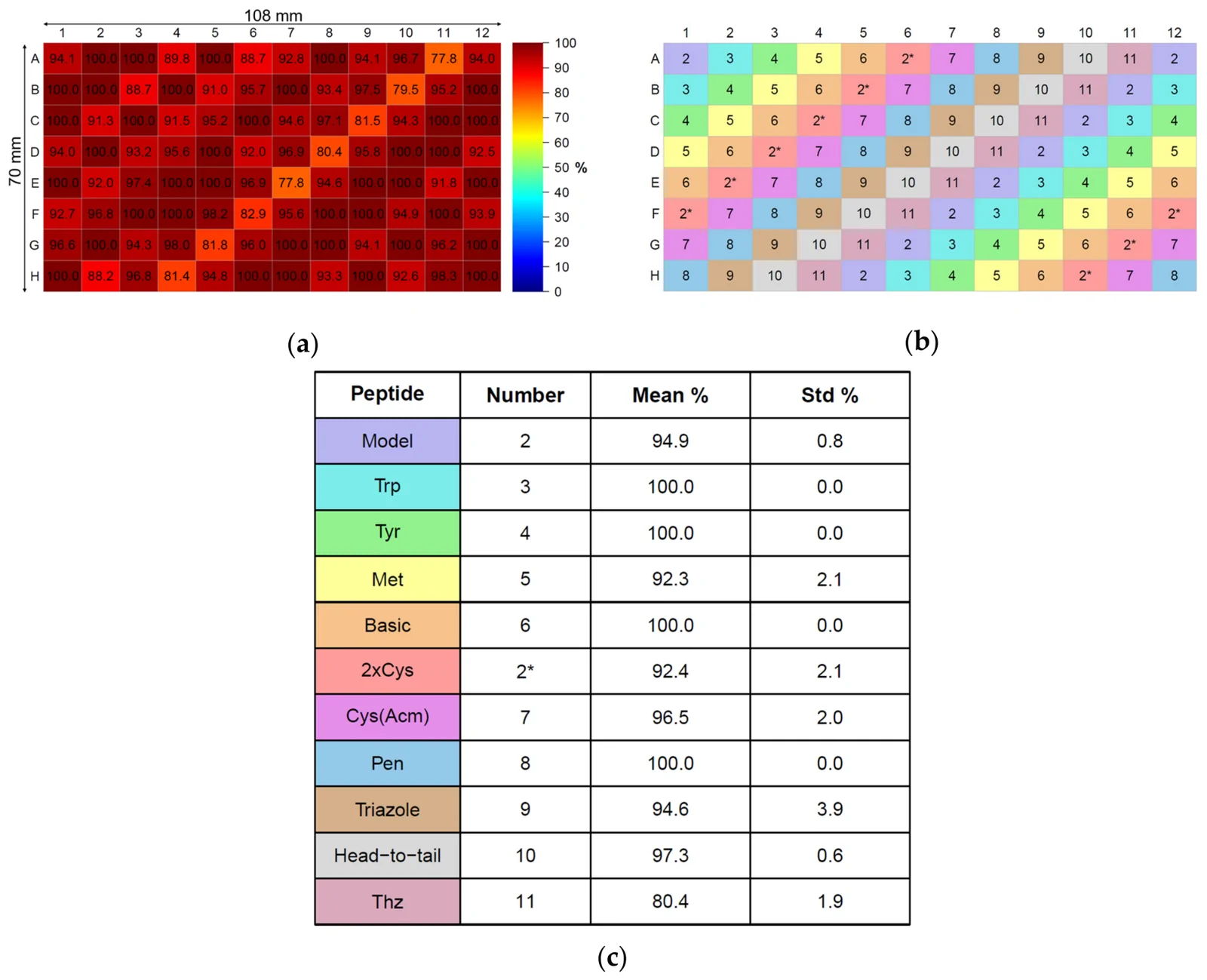
(**a**) Heatmap of percentage of product formation of combinatorial peptide library after 130 min irradiation 50 mm from the array (*n* = 1). (**b**) Layout of peptide substrates in the 96-well plate. (**c**) Conversion of peptide substrates in plate format. 2* indicates a peptide identical to **2** resulting from desulfurization of two Cys residues. Formation of peptide **3** includes oxidized product (8.9 ± 1.5%). Formation of peptide **11** includes Thz deprotection and double desulfurization (18.3 ± 3.6%).

Following optimization of the plate-based desulfurization conditions using the model peptide, a combinatorial peptide library was subjected to the optimized reaction conditions in a 96-well format. High conversion was observed across the entire plate, and wells containing identical peptide substrates showed comparable conversions, confirming the reproducibility of the protocol (Figure 5). Peptides exhibiting slower reaction kinetics were readily identified, with peptide **11** representing a notable example. These results highlight the capability of the LED array to facilitate desulfurization in high-throughput synthesis of combinatorial peptide libraries.

### 2.5. Perspectives and Outlook

Uniform irradiation across the 96-well plate was found to be essential for ensuring uniform product formation in all wells. The reduced reaction rate observed in buffers containing MeCN may prolong reaction time in plate format of libraries comprising peptides with lower solubility than the substrates tested in this study. However, this could potentially be mitigated by employing degassed solvents.

The presented protocol has several practical advantages compared to existing desulfurization strategies. It enables one-pot ligation and desulfurization without external hydrogen atom sources, radical initiators, photoredox catalysts, or photosensitizers, in a multi-well format, highlighting the operational simplicity of the strategy.

In contrast to protocols employing LiBEt_3_H, NaBH_4_ or NaBEt_4_, the current method eliminates the use of pyrophoric reagents and does not require buffer acidification prior to the addition of the radical initiator. This highlights the current strategy as a “hands-off” method and simplifies the experimental workflow, reduces handling complexity, and improves operational safety, particularly in high-throughput formats.

Beyond desulfurization, LED arrays/lamps may enable a broader range of photochemical peptide transformations in plate format, offering an operationally simple approach for high-throughput synthesis of combinatorial peptide libraries [37].

## 3. Materials and Methods

### 3.1. Chemicals and Reagents

Protected amino acids were purchased from Iris Biotech GmbH (Marktredwitz, Germany) and Ambeed, Inc. (Buffalo Grove, IL, USA). SPPS reagents were purchased from Iris Biotech and Sigma Aldrich (St. Louis, MO, USA). Buffer components were purchased from Sigma Aldrich. Dichloromethane, acetonitrile, and diethyl ether were purchased from VWR (Radnor, PA, USA); TFA was purchased from Iris Biotech. All water was milliQ from Biopak filter (Merck KGaA, Darmstadt, Germany). Water used in LCMS buffer was milliQ from LC-Pak filter (Merck KGaA).

### 3.2. Instruments

All peptides were synthesized on a Liberty Blue 2.0 Microwave Peptide Synthesizer (CEM Corporation, Matthews, NC, USA) on a 0.2 mmol scale. All peptides were purified using a high-performance liquid chromatography (HPLC) system (Waters Corporation, Milford, MA, USA) comprising a Waters 2545 quaternary gradient module, a Waters 2707 autosampler, a Waters 2489 UV/vis detector, and a Waters fraction collector III, equipped with a preparative C18 column (ReproSil pHoenix, C18, 100 Å, 5 μm, 30 mm × 250 mm; Dr. Maisch HPLC GmbH, Ammerbuch, Germany). Peptides were quantified using ultra-performance liquid chromatography charged aerosol detector (UPLC-CAD) instruments (ThermoFisher Scientific, Waltham, MA, USA) comprising a Vanquish detector, autosampler, pump, and an analytical C18 column (Luna Omega, C18, 100 Å, 1.6 μm, 2.1 mm × 50 mm; Phenomenex, Torrance, CA, USA). UPLC-CAD data analysis was performed using Chromeleon Chromatography Data System (v7.2.10 ES, ThermoFisher Scientific). All samples were analyzed using a Waters ACQUITY LCMS instrument (Waters Corporation) comprising a TUV detector, sample manager—FTN, quaternary solvent manager, and an SQ detector 2, equipped with a 3100 Mass Detector, separated using an analytical C18 column (ACQUITY UPLC Peptide CSH, C18, 130 Å, 1.7 μm, 2.1 mm × 100 mm; Waters Corporation). LCMS data analysis was performed using MassLynx Software (v4.2, SCN985, Waters Corporation). Single samples were irradiated using single LEDs (M265L5 and M275L4) and a T-Cube LED Driver (LEDD1B) (Thorlabs, Inc., Newton, NJ, USA). The single LEDs were mounted into a metal box with inner dimensions of 6.8 cm × 7.1 cm × 9.5 cm and a circular hole with diameter of 2.5 cm for the LED.

### 3.3. Peptide Synthesis and Purification

All peptides were synthesized on TentaGel S RAM resin with a loading capacity of 0.24 mmol/g. Deprotection of the resin was carried out with 10% piperidine/0.1 M 1-hydroxybenzotriazole (HOBt)/*N*,*N*′-dimethylformamide (DMF) for 1 min at 90 °C followed by four washing steps with DMF. Coupling was performed using 0.2 M Fmoc-amino acid with 0.2 M ethyl 2-cyano-2-(hydroxyamino)acetate (OxymaPure)/DMF in 5-fold excess to the resin, activated by 1.0 M *N*,*N*′-diisopropylcarbodiimide (DIC) in 10-fold excess to the resin, coupled for 2 min at 90 °C. Arginine residues were subjected to double coupling. Following peptide assembly, all resins were washed with DMF and dichloromethane (DCM).

Peptide **10** was synthesized with a C-terminal *N*-acyl-*N*′-methyl-benzimidazolinone (MeNbz) linker for cyclization. Prior to the assembly of the desired peptide sequence, glycine was coupled to the resin followed by Fmoc-MeDbz-OH (3,4-diaminobenzoic acid) using a preactivated mixture of 4 equiv. Fmoc-MeDbz-OH, 3.9 equiv. 2-(1H-7-azabenzotriazol-1-yl)-1,1,3,3-tetramethyluronoium hexafluorophosphate (HATU), and 8 equiv. DIPEA in DMF. Double coupling was performed for 2 h at room temperature (RT). Coupling of the subsequent amino acids was performed as described above. Coupling of the N-terminal cysteine was performed using a Boc-protected α–amine. Acylation of MeDbz was performed with two treatments of 50 mM 4-nitrophenyl chloroformate/DCM for 30 min at RT, followed by washing with DCM and DMF after the final acylation. MeNbz formation was performed using two treatments of 0.25 M DIPEA/DMF for 15 min at RT, followed by washing with DMF and DCM for the final wash.

Peptides were deprotected and cleaved from the resin using 92.5% trifluoroacetic acid (TFA)/2.5% water/2.5% triisopropylsilane (TIPS)/2.5% 2,2′-(ethylenedioxy)diethanethiol (DODT) (*v*/*v*) for 2.5 h at RT. The peptides were precipitated in cold diethyl ether and centrifuged at 4400 rpm for 2 min. Precipitation was performed twice and residual ether was evaporated overnight. The peptides were then dissolved in 50% MeCN (*v*/*v*) and lyophilized.

For head-to-tail cyclization of peptide **10** by NCL, the peptide was dissolved in a minimal amount of 50% MeCN (*v*/*v*) required for dissolution. The peptide mixture was added dropwise to a 100 mM phosphate buffer at pH 7 while stirring and heating at 50 °C for a final peptide concentration of 4 mg/mL. After 20 min, LCMS confirmed that all starting material had been cyclized. The solution was acidified with TFA prior to purification by HPLC.

For the intramolecular copper-catalyzed azide alkyne cycloaddition on peptide **9**, the peptide was dissolved in a 50 mM phosphate buffer at pH 7 with 30% MeCN (*v*/*v*) for a peptide concentration of 0.3 mg/mL. A premixed solution of 100 mM CuSO_4_ (1.4 equiv.) and 100 mM tris(3-hydroxypropyltriazolylmethyl)amine (THPTA) (7 equiv.) was prepared in a separate container and added to the peptide solution. A solution of 100 mM sodium ascorbate (5 equiv.) was added to start the reaction. The mixture was stirred and heated at 40 °C in a closed container under N_2_ atmosphere. After 1 h, LCMS confirmed that all starting material was consumed. The solution was acidified with TFA prior to purification by HPLC.

For purification by HPLC, the peptides were dissolved in 10% MeCN (*v*/*v*) and purified using a mobile phase consisting of solvent A (0.1% TFA/water) and solvent B (0.1% TFA/MeCN). A gradient of 10–35% B in 30 min with a flow rate of 20 mL/min and a fraction volume of 10 mL was used. Fractions were analyzed by LCMS using a gradient of 15–35% B in 12 min.

Pooled fractions were analyzed by UPLC-CAD using a mobile phase consisting of solvent A (0.1% formic acid/water) and solvent B (0.1% formic acid/MeCN). A gradient of 0–80% B in 3.5 min with a flow rate of 10 mL/min was used. The concentration was determined using a standard curve generated from increasing injection volumes of NovoRapid (Novo Nordisk, Bagsværd, Denmark).

### 3.4. Light-Mediated Desulfurization in Single Samples

**Figure 6:**
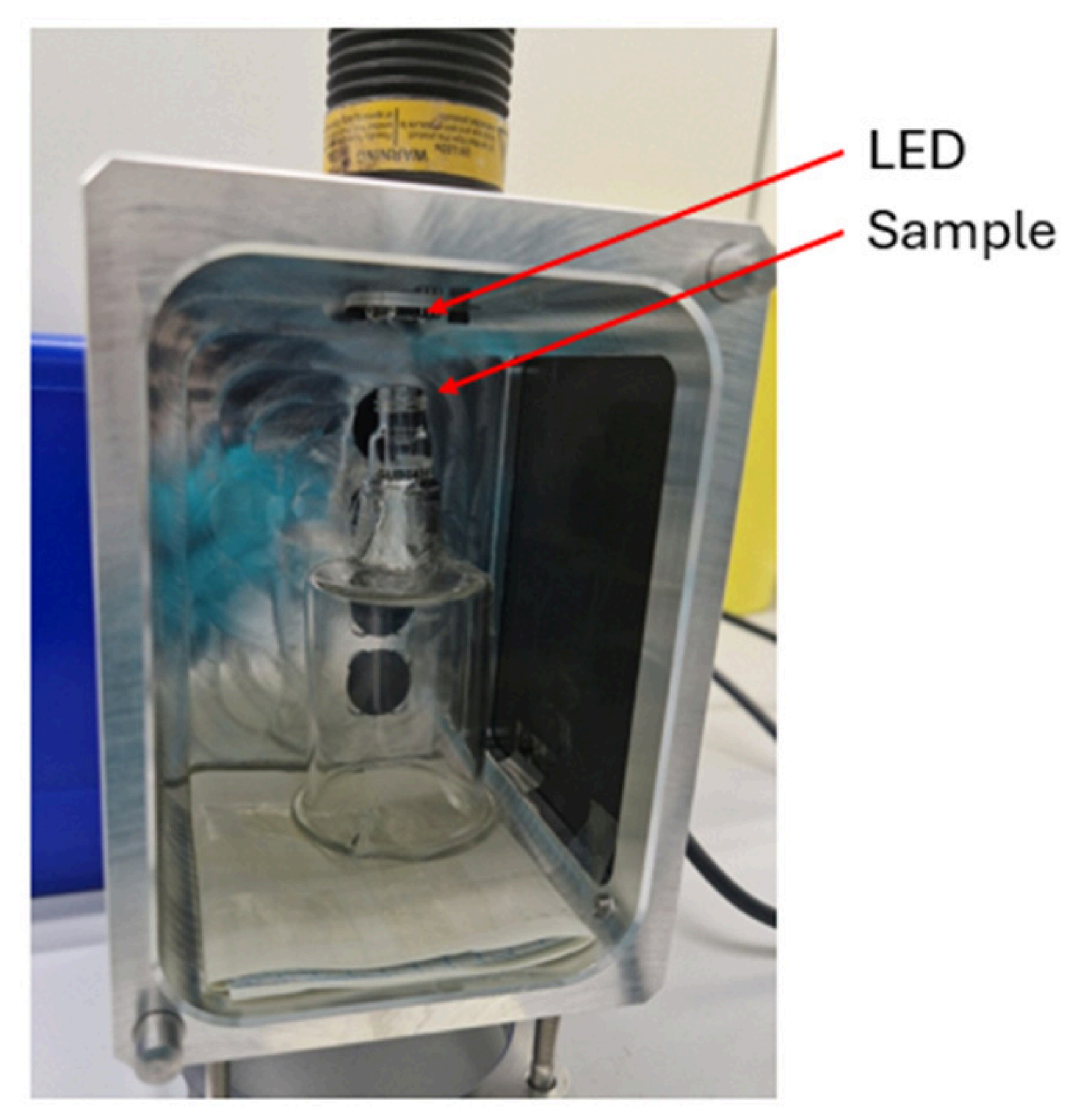
Experimental set-up for irradiation of single samples. The sample is in the inlet of a glass LCMS vial.

Aliquots of 0.221 μmol peptide were dissolved in 250 μL desulfurization buffer and 40 μL were transferred to LCMS vials. The vials were uncapped and positioned 35 mm from the LED and irradiated one at a time from above. The vial and LED were positioned inside a metal box (Figure 6) with a lid to prevent light from entering or exiting. Samples were irradiated for 30 min at maximum current. After irradiation, 20 μL of 100 mM HQ or mQ were added to the samples. Samples were analyzed by LCMS using a gradient from 20 to 40% B in 12 min and the same column as described previously.

For samples analyzed using an internal standard, 60 μL of reference compound were added before LCMS analysis. The reference compound used for linear peptide **1** was a 0.0493 mM solution of a peptide with sequence IKPEAPGEDASPEELNR-YYASLRHYLNLVTRQRY-NH_2_ in 50% MeCN (*v*/*v*). The reference compound used for head-to-tail cyclic peptide **10** was a 0.0533 mM solution of a peptide with sequence IKPEKeps(C18DA-gGlu)PGEDASPEELNRYYASLRHYLNLVTRQRY-NH_2_ in 50% MeCN (*v*/*v*).

Degassing was performed by bubbling N_2_ through the peptide solution for 1 min followed by bubbling N_2_ through the vial for 1 min. After transferring 40 μL to LCMS vials, these vials were degassed again for 1 min.

### 3.5. Chemical Desulfurization with NaBEt_4_

Aliquots of 0.221 μmol peptide were dissolved in 250 μL 0.1 M citric acid buffer with 10% MeCN (*v*/*v*) and pH 4. Aqueous solutions of TCEP (5 equiv.) and NaBEt_4_ (20 equiv.) were added and mixed. For the control samples, mQ was added instead of NaBEt_4_.

### 3.6. Light-Mediated Desulfurization in 96-Well Plate

**Figure 7:**
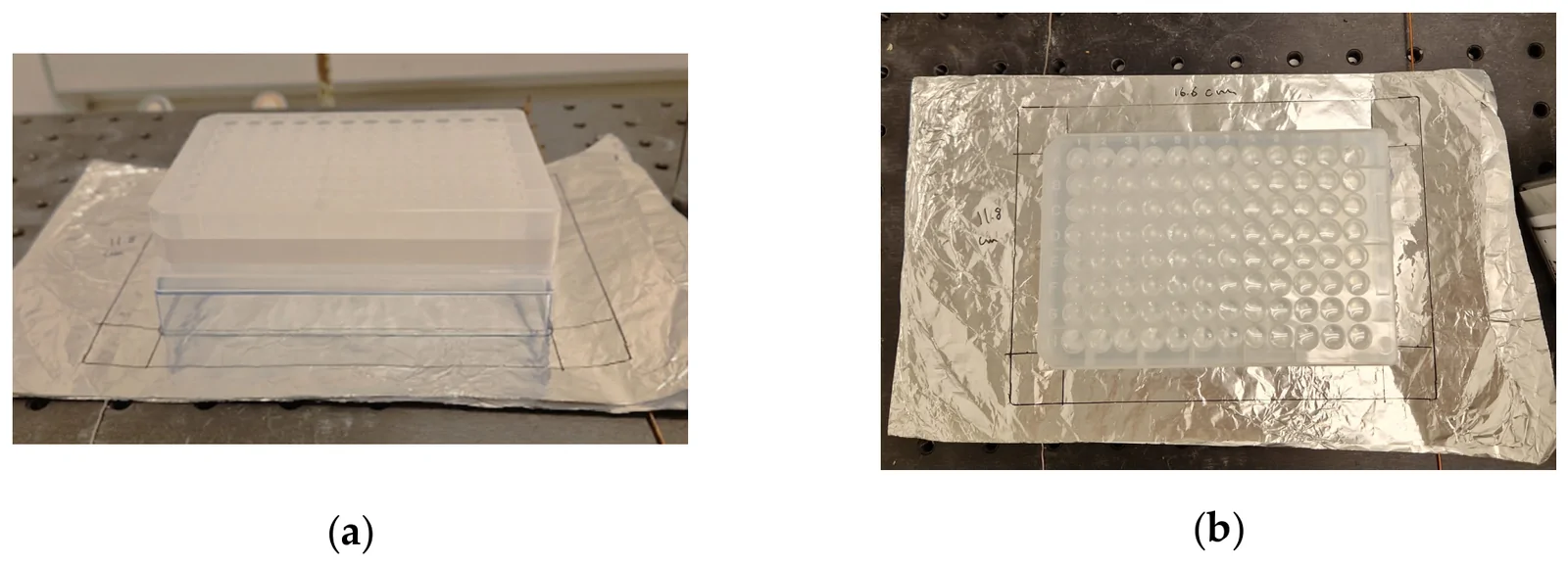
Experimental set-up for irradiation of 96-well plate: (**a**) side view; (**b**) top view. Lines were drawn on the foil to align the center of the LED array with the well plate.

In a polypropylene 96-well plate with a working volume of 500 μL per well (BRAND GMBH + CO KG, Wertheim, Germany), 0.43 μmol of peptide **1** (0.86 μmol/mL) were dissolved in 0.1 M PB with 8.8 mM TCEP at pH 7 in each well (Figure 7). The plate was centered under the lamp at a vertical distance of 35, 42.5, or 80 mm from the LED array (Figure S13). The first-generation UV LED lamp system was fitted with a 4 × 3270 nm LED array, which was operated in series at 18.14 V and 1.190 A, yielding a center irradiance of 8.00 mW/cm^2^ at 82.5 mm distance.

The experiment was repeated using a second-generation lamp. This second-generation UV LED lamp system was fitted with a 4 × 3 plus 3 × 2270 nm UV LED array, which was operated at 18.78 V and 2.013 A, yielding a center irradiance of 13.65 mW/cm^2^ at 82.5 mm distance. The deep well plate was centered 35 or 50 mm from the LED array. The same conditions were employed for desulfurization of the combinatorial peptide library in plate format.

For temperature monitoring, four plastic vials containing water (2 mL per vial) were evenly placed under the second-generation lamp at a 35 mm distance using the same lamp operating settings described above. The temperatures of the irradiated water and a non-irradiated reference water were recorded six times over a 130 min period, showing temperature increases of up to 5°C (Figure S31).

### 3.7. NMR Analysis of Model Peptide and Product

The NMR samples were dissolved in 180 μL DMSO-*d6* and transferred to 3 mm NMR tube. All 1D and 2D NMR spectra were recorded at 298 K on a Bruker AVANCE III 800 MHz spectrometer equipped with a 5 mm TCI CryoProbe (Bruker BioSpin GmbH, Rheinstetten, Germany), using standard pulse sequences and Non Uniform Sampling (NUS) for the 2D homonuclear ^1^H–^1^H DQF-COSY and TOCSY spectra, ^1^H–^13^C edHSQC spectra and ^1^H–^15^N HSQC spectra. 2D NOESY spectra were acquired with a mixing time of 600 ms. The ^1^H and ^13^C NMR chemical shifts are reported with reference to the residual solvent signals at *δ*_H_ 2.49 and *δ*_C_ 39.5 ppm for DMSO-*d6*. NMR data were processed and analyzed using Bruker Topspin 4.3.1 (Bruker BioSpin GmbH). Overview of the 1D and 2D NMR spectra and tables with the resonance assignments of ^1^H, ^15^N and some of the ^13^C resonances for both the model peptide **1** and the product **2** can be found in the Supplementary Section (Tables S1 and S2, Figures S32–S43). Reference [38] is cited in the Supplementary Materials.

### 3.8. Peak Analysis from UV Trace 220 nm by LCMS

For experiments using hydroquinone, calculations of product and starting material hydroquinone adduct (SM-HQ) percentage were done as follows.

(1) $$Y_{Product,hydroquinone}=A_{product}/(A_{product}+A_{\mathrm{SM}}+A_{\text{SM-HQ}})\times 100\%,$$

(2) $$Y_{SM,hydroquinone}=A_{\mathrm{SM}}/(A_{product}+A_{\mathrm{SM}}+A_{\text{SM-HQ}})\times 100\%.$$

For experiments not using hydroquinone, calculations of product and starting material percentages were done as follows.

(3) $$Y_{Product}=A_{product}/(A_{product}+A_{\mathrm{SM}})\times 100\%,$$

(4) $$Y_{SM}=A_{\mathrm{SM}}/(A_{product}+A_{\mathrm{SM}})\times 100\%.$$

*A*_product_, *A*_SM_, and *A*_SM-HQ_ denote peak area of product, peak area of starting material, and peak area of the presumed starting material hydroquinone adduct, respectively, from the 220 nm UV trace acquired using LCMS. For samples analyzed using an internal standard compound, calculations were done as follows.

(5) $$\mathrm{Conversion}\;\mathrm{by}\;\mathrm{light}\;X_{conversion,light}=\left(\frac{A_{product}+A_{SM}/A_{std}}{A_{SM,\;control}/A_{std}}\right)\times 100\%,$$

(6) $$\mathrm{Conversion}\;\mathrm{by}\;{\mathrm{NaBEt}}_{4}\;X_{conversion,NaBEt4}=\left(\frac{A_{product}+A_{SM}/A_{std}}{A_{SM,\;control}/A_{std}}\right)\times 100\%.$$

*A*_std_ represents peak area of the internal standard compound and *A*_SM,control_ represents the starting material peak area of a control sample (0 min irradiation or exchanging NaBEt_4_ with mQ).

### 3.9. Lamp Measurements and Irradiance Heatmap Calculations

The spectral irradiance of the two single LEDs (M265L5 (265 nm) and M275L4 (275 nm), Thorlabs) were measured using an integrating sphere spectroradiometer setup. It consists of an integrating sphere (ISP150UV-100, Instrument Systems, Munich, Germany) that has an input port with a diameter of 25 mm, and the sphere was fiber-coupled to a spectroradiometer (CAS 140D, Instrument Systems). It was calibrated to total spectral flux from 200 nm to 830 nm. For spectral irradiance, the measured spectral flux is divided by the area of the input port. The LEDs were mounted at a distance of 80 mm from the input port of the sphere perpendicular to the port. Spectral irradiance measurements have been made as a function of operating current, from 50 mA up to the maximum permissible current for the individual LEDs.

A large area UV LED lamp was designed and custom built to yield a homogeneous and high irradiance across a multi-well plate format (108 mm × 70 mm), as seen in the photo in Figure S13c. The lamp has a dimension of 150 mm × 100 mm and 12 UVC LEDs (UVC LED S3535-H-DR350-265nm, BOLB Inc., Livermore, CA, USA) are individually mounted on 10 mm × 10 mm PCBs, and they are mounted as a 4 × 3 array with 4 LEDs along the long direction and 3 LEDs along the short direction on a heatsink as seen in the photo in Figure S13a. The corresponding distances between the LEDs are 37.5 mm and 33.3 mm, respectively. Walls of 80 mm height with sheets of polytetrafluoroethylene (PTFE) (PMR10P1, Thorlabs) provide high diffuse reflection of more than 93% and make up the outer sides of the lamp. The well plate was placed within the walls with distances to the LEDs ranging from 35 mm to 80 mm.

A total spectral flux of a single LED has been characterized as a function of operating current at 25 °C using the integrating sphere spectroradiometer, producing a total flux of 188 mW with an efficacy of 5.9% at the maximum current of 500 mA. The LED operating current in the experiments was approximately 300 mA, and the corresponding flux was around 100 mW per LED in the lamp. The peak wavelength and centroid wavelength were 270.4 nm and 273.3 nm, respectively, and the spectral width (full width at half maximum, FWHM) was 10.4 nm. This version of the LED lamp is called the first-generation lamp.

Electrically, the LEDs were connected as four parallel connections each with three LEDs in series with a voltage up to approximately 18.5 V. Due to the low efficacy of around 6% at LED operating currents from 300 mA to 400 mA, heat generation was up to 36 W. Therefore, a fan was installed to force air cooling of the heatsink and keep the operating temperature of the heatsink below 45 °C. The LEDs have a hemispherical lens and a resulting angular distribution with a FWHM value of 70°.

To generate higher and more homogeneous irradiation of the well plate area, a second-generation LED lamp was produced by adding six extra LEDs to the first-generation lamp as a 3 × 2 array of LEDs with distances between the LEDs of 37.5 mm and 33.3 mm, hence filling the spaces in the 4 × 3 array of LEDs (Figure S13b). The second-generation lamp has 18 LEDs connected as six parallel connections each with three LEDs in series and the voltage is the same as for the first-generation lamp.

A combination of spectral irradiance measurements and a simulation model of the LED lamps was used to characterize the irradiance distribution in the planes and distances from the LEDs where the well plate was positioned in the experiments.

For the spectral irradiance measurement, the 25 mm port of the integrating sphere was placed 2.5 mm in front of the walls and along the central or optical axis of the lamp, hence 82.5 mm from the LEDs. For both generations of the lamp, this central irradiance was measured as a function of the operating current (Figure S13d).

A simulation model of the UV LED lamps was set up using Zemax OpticStudio (#27330, Ansys, Canonsburg, PA, USA) in non-sequential mode. A 3D model of the lamp was created and the emission from the individual LEDs was simulated using a rayfile for a similar LED with the same angular distribution (XST-3535-Zx_5m.dat, Luminus, Sunnyvale, CA, USA). The PTFE sheet walls were simulated using diffuse reflection. Rectangular detectors of size 132 mm × 90 mm were placed perpendicular to the optical axis at distances of 35, 42.5, 50, 60 and 80 mm from the LEDs. The detectors have 65 × 45 pixels with an approximate size of 2 mm × 2 mm. Further, a ø25 mm circular detector surface is placed at 82.5 mm to simulate the response of the irradiance measurements using the 25 mm integrating sphere port.

Simulations of the irradiance on the detectors were performed using raytracing of 1 million rays from each of the LEDs. The total flux of the individual LEDs was set at a simulated value so that the simulated ø25 mm detector matched that of the measured central irradiance at 82.5 mm for the specific operating current of the lamp. The first-generation lamp was operated at a current of 1.190 A, which corresponds to a central irradiance at 82.5 mm of 8.00 mW/cm^2^. The total flux per LED was 100 mW, corresponding to an LED current of 298 mA. For this setting of the first-generation lamp, irradiance distributions were calculated at the operating distances of 35, 42.5 and 80 mm. For the second-generation lamp, the operating current was 2.013 A, which corresponds to a central irradiance at 82.5 mm of 13.65 mW/cm^2^. The total flux per LED was 106 mW, corresponding to an LED current of 336 mA. For this setting of the second-generation lamp, irradiance distributions were calculated at the operating distances of 35, 42.5, 50, 60 and 80 mm. The operating current and corresponding central irradiance of the two UV lamps are shown in Figure S13d.

Simulated 2D irradiance distributions were exported from Zemax OpticStudio (65 × 45 spatial matrix, active aperture of 132 mm × 90 mm, detector distance set at Z = 50.00 mm) and visualized using custom R2022b MATLAB scripts (MathWorks, Inc., Natick, MA, USA). Simulated irradiance values were scaled from W/cm^2^ to mW/cm^2^ and plotted as continuous spatial heatmaps using imagesc function with a fixed jet colormap range and compared directly with product formation heat maps across the 96-well plate (Figure 3b–d and Figure 4b).

To determine the irradiance exposure within individual wells of a standard 96-well plate (108 × 70 mm, 8 × 12 matrix, 9.0 mm center-to-center pitch), a 2D spatial mesh grid corresponding to the center coordinates of each well (*X_well_*, *Y_well_*) was generated across the detector area. The data were smoothed using a 2D Gaussian filter (imgaussfilt, σ = 3) to suppress residual stochastic noise while maintaining true spatial gradients. Bilinear 2D spatial interpolation (interp2) was applied to sample the smoothed irradiance at the center coordinates of all 96 individual wells. The discretized well irradiance was then visualized on a matching spatial grid to allow direct spatial comparison against experimental product formation heatmaps (Figures S17 and S22).

Additional transmission measurements were carried out on relevant liquid sample holders and well-plate materials to assess how much UV light was attenuated before reaching the samples (Figure S20).

### 3.10. Use of Generative Artificial Intelligence

Generative artificial intelligence (ChatGPT v5.5, OpenAI) was used to create illustrative figures included in this manuscript. The generated figures were subsequently reviewed and edited by the authors. The figures are only intended for visualization and do not contain any data.

## 4. Conclusions

We have developed an operationally simple desulfurization strategy employing an LED array to enable the desulfurization of multiple peptide substrates simultaneously in plate format. Using UV light, no photocatalysts or radical initiators were necessary to drive the reaction, and the only reagent employed in the buffer system was a water-soluble phosphine reagent. The optimized reaction conditions entailed a wavelength of ~270 nm and the use of 10 equiv. of TCEP, affording the fastest conversion. The new protocol proved applicable with peptides containing various functional groups, demonstrating the robustness of the method. In plate format, product formation was directly correlated with local light exposure revealing the importance of uniform irradiance to ensure homogenous product formation. This is the first example of light-mediated desulfurization of combinatorial peptide libraries in plate format reported in the literature. The use of LED arrays for the photochemical modification of peptides has potential applications beyond desulfurization and may enable a broader range of reactions facilitating high-throughput synthesis of combinatorial peptide libraries for drug discovery.

## Figures and Tables

**Table 1:** Peak area percentage of remaining peptide corresponding to product after 30 min irradiation (Figures S2–S9). All results are presented as the mean of three samples, *n* = 3. PB: phosphate buffer.

Buffer	Wavelength (nm)	TCEP (eq.)	pH	MeCN (*v*/*v* %)	Product (%) ± SD
0.1 M PB	265	0	7	0	4.3 ± 1.2
0.1 M PB	265	2.5	7	0	34.9 ± 2.8
0.1 M PB	265	5	7	0	78.0 ± 2.8
0.1 M PB	265	10	7	0	91.9 ± 1.9
0.1 M PB	265	20	7	0	95.0 ± 1.1
0.1 M PB	275	20	7	0	52.5 ± 10.8
MeCN/PB	265	10	7	25	44.4 ± 3.1
MeCN/PB	265	10	7	50	23.1 ± 3.2
0.1 M PB ^a^	265	10	7	0	92.4 ± 1.6
MeCN/PB ^a^	265	10	7	25	27.0 ± 8.2
MeCN/PB ^a^	265	10	7	50	29.7 ± 2.8
0.1 M PB ^b^	265	5	7	0	86.0 ± 2.8
MeCN/PB ^b^	265	10	7	25	68.1 ± 5.8
MeCN/PB ^b^	265	10	7	50	53.9 ± 3.6
0.1 M PB	265	5	6	0	16.7 ± 1.5
0.1 M PB	265	5	8	0	71.5 ± 2.9
0.1 M Tris	265	5	7	0	51.7 ± 2.1
0.1 M Tris	265	5	8	0	62.6 ± 11.5
0.1 M Tris	265	5	9	0	73.2 ± 3.4
MeCN/Tris	265	5	7	0	9.0 ± 6.5
MeCN/Tris	265	5	8	25	8.8 ± 2.4
MeCN/Tris	265	5	9	25	8.2 ± 3.1

^a^ 10 equiv. DIPEA. ^b^ Degassed with nitrogen prior to irradiation.

## Data Availability

The data supporting the findings of this article are available from the corresponding authors upon reasonable request.

## Supplementary Materials

The following supporting information can be downloaded at: https://www.mdpi.com/article/10.3390/molecules31173097/s1.
