# Supplementary material for: Light-Mediated Desulfurization of Peptides in Multi-Well Format for Combinatorial Library Synthesis
Source: Molecules. 2026 Sep 3;31(17):3097. doi: 10.3390/molecules31173097 (PMC13566937; doi:10.3390/molecules31173097)

Supporting information
Light-Mediated Desulfurization of Peptides in Multi-Well Format for Combinatorial Library Synthesis

Esther Arentoft Jacobsen [1,2], Julian Lava [2], Anders Thorseth [3], Dennis Dan Corell [3], Carsten Dam-Hansen [3], Charlotte Held Gotfredsen[2], Martin Kræmer[1], Poanna Tran [1,*], Katrine Qvortrup [2,*]

[1]Gubra, Hørsholm 2970, Denmark;
[2]Department of Chemistry, Technical University of Denmark, Kongens Lyngby 2800, Denmark;
[3]Department of Electrical and Photonics Engineering, Technical University of Denmark,Kongens Lyngby 2800, Denmark;

*Correspondence: ptr@gubra.dk (P.T.); kaqvo@kemi.dtu.dk (K.Q.)

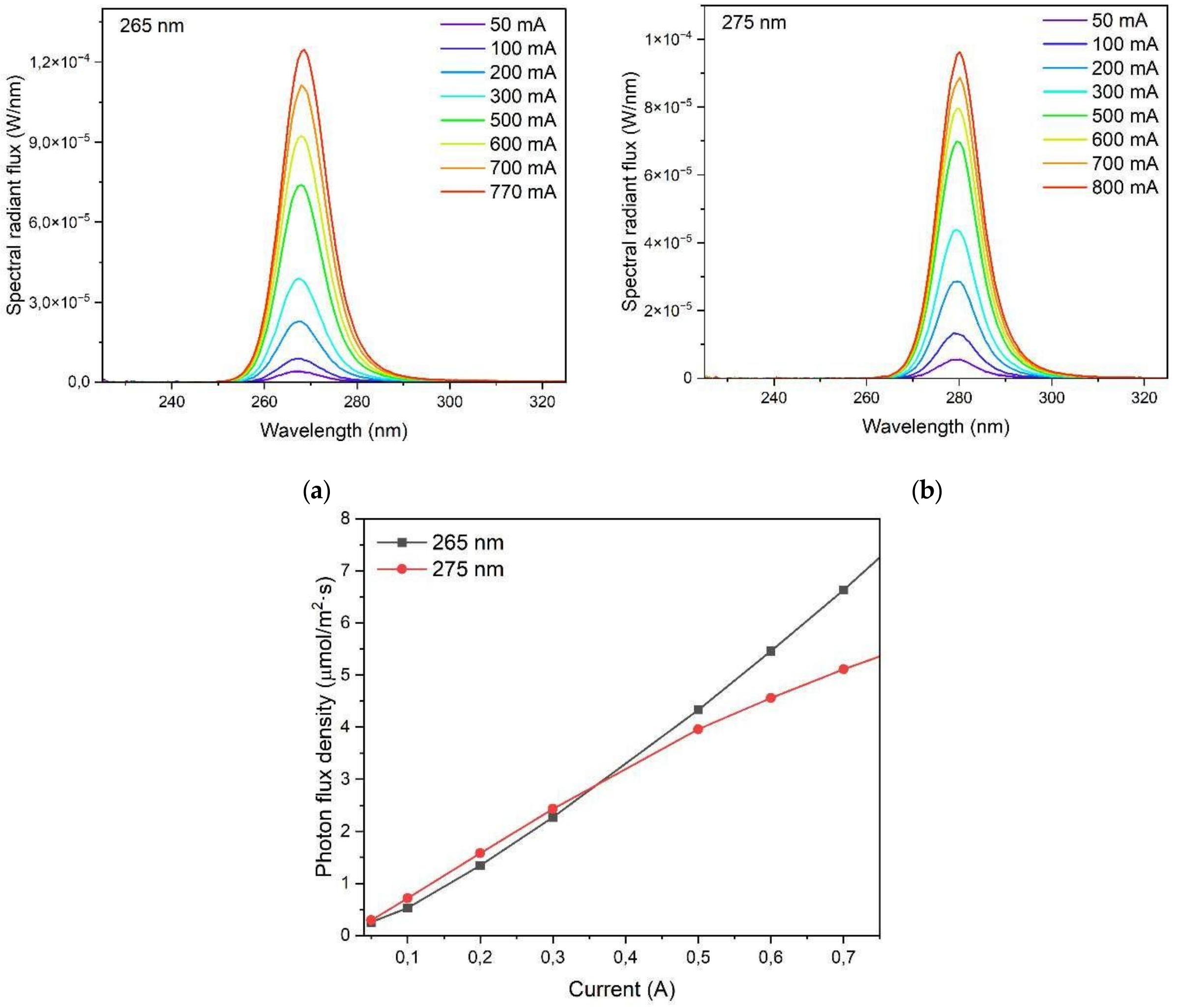

(a) (b)

(c)

Figure S1. Spectral characteristics and current-dependent photon flux density of Thorlabs M265L5 (265 nm) and M275L4 (275 nm) LEDs. The measured spectral radiant flux as a function of wavelength across various driving currents are shown for the (**a**) 265 nm and (**b**) 275 nm LEDs. (**c**) Resulting photon flux density, calculated from the spectral data, plotted as a function of the driving current.

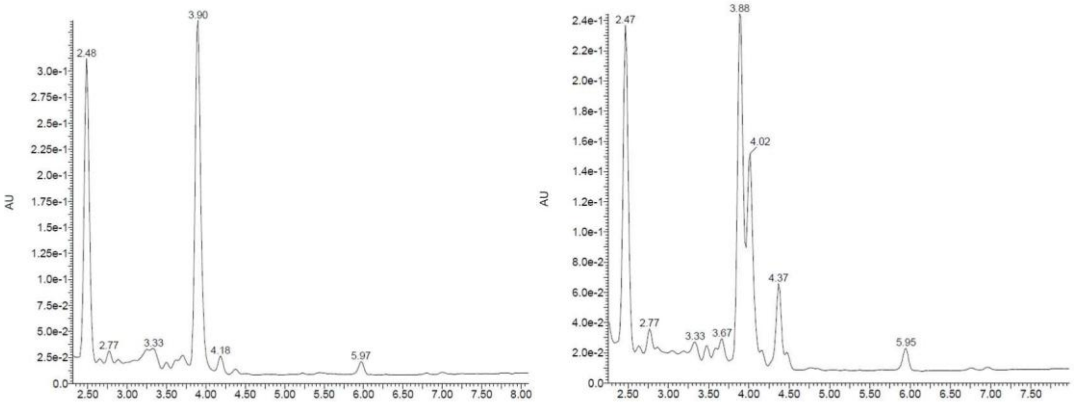

(**a**) (**b**)

Figure S2. UV traces from LCMS for optimization of wavelengths after 30 min irradiation. Gradient: 20–40% B over 12 min.
(**a**) Sample irradiated with 265 nm. Product displays retention time (RT) = 3.90 min. (**b**) Sample irradiated with 275 nm. Product displays RT = 3.88 min, SM displays RT = 4.02 min, and presumed SM-hydroquinone (SM-HQ) adduct displays RT = 4.37 min.

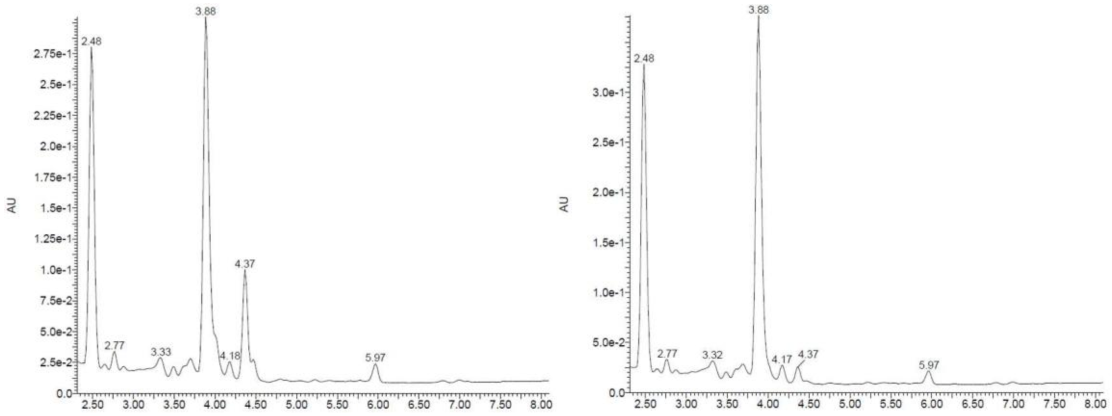

(**a**) (**b**)

Figure S3. UV traces from LCMS for optimization of TCEP concentrations. Gradient: 20–40% B over 12 min. (**a**) Sample with 5 equiv. TCEP. Product displays RT = 3.88 min, SM (right shoulder on product peak) displays RT = 4.01 min, and SM-HQ adduct displays RT = 4.37 min. (**b**) Sample with 10 equiv. TCEP. Product displays RT = 3.88 min and SM-HQ adduct displays RT = 4.37 min.

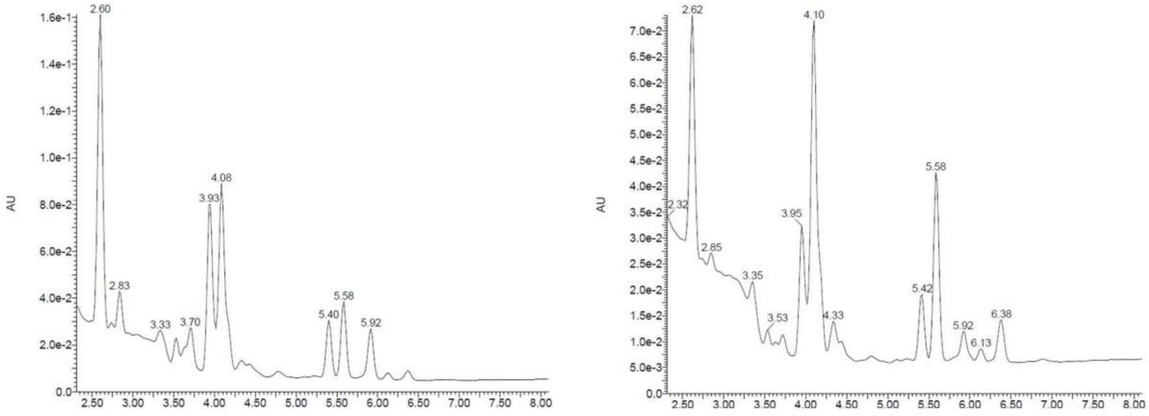

(**a**) (**b**)

Figure S4. UV traces from LCMS containing MeCN. Gradient: 20–40% B over 12 min. (**a**) Sample with 25% MeCN. Product displays RT = 3.93 min and SM displays RT = 4.08 min. (**b**) Sample with 50% MeCN. Product displays RT = 3.95 min and SM displays RT = 4.08 min.

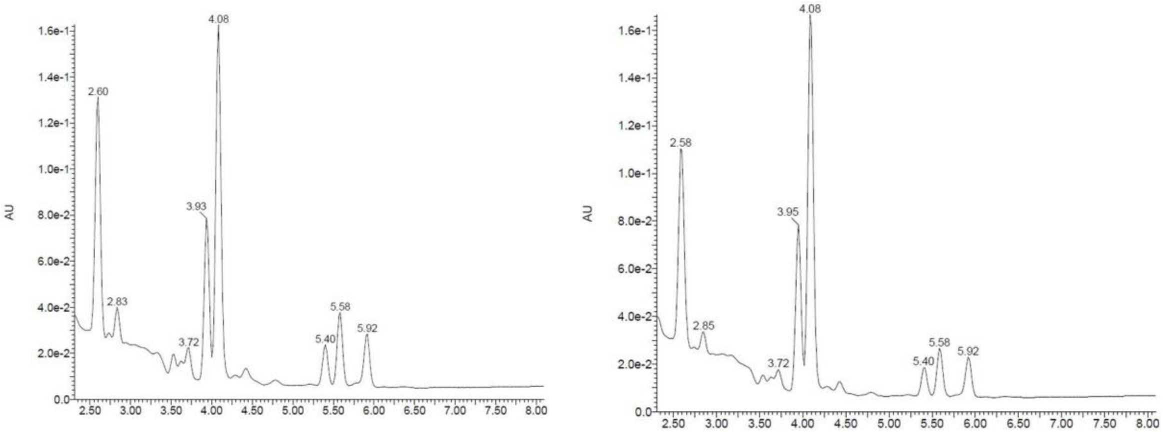

(**a**) (**b**)

Figure S5. UV traces from LCMS containing MeCN and DIPEA. Gradient: 20–40% B over 12 min. (**a**) Sample with 25% MeCN. Product displays RT = 3.93 min and SM displays RT = 4.08 min. (**b**) Sample with 50% MeCN. Product displays RT =
3.95 min and SM displays RT = 4.08 min.

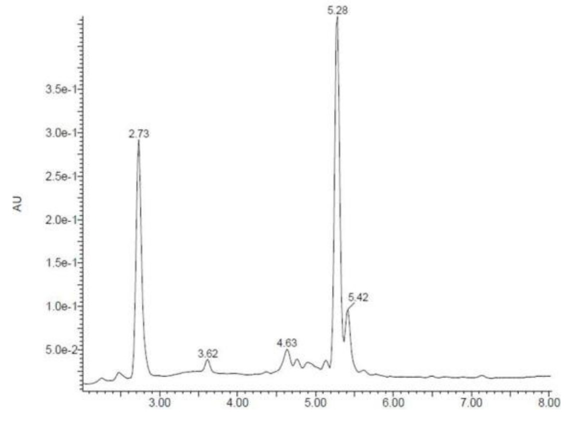

(**a**)

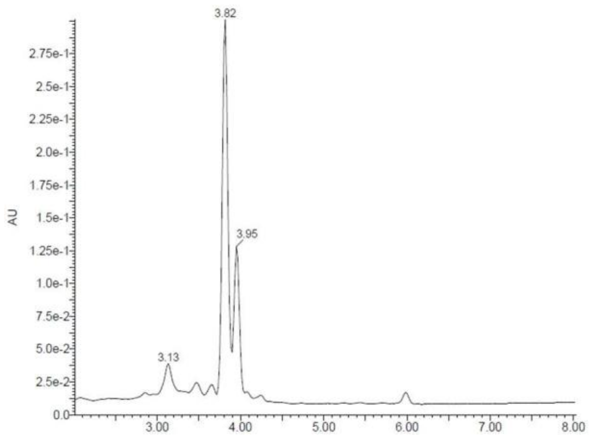

(**b**)

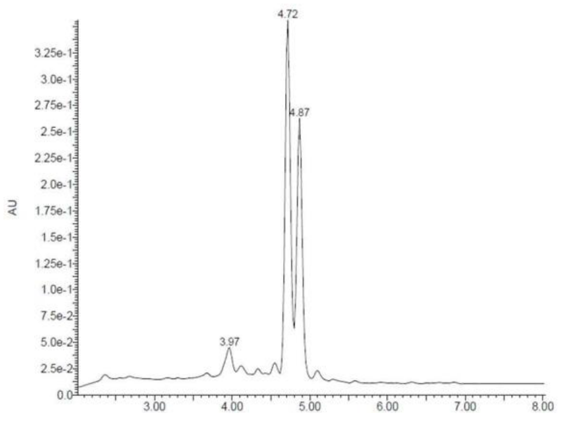

(**c**)

Figure S6. UV traces from LCMS containing degassed buffers. Gradient: 20–40% B over 12 min. (**a**) Sample with 5 eq. TCEP, no MeCN. Product displays RT = 5.28 min and SM displays RT = 5.42 min. (**b**) Sample with 10 eq. TCEP and 25% MeCN. Product displays RT = 3.82 min and SM displays RT = 3.95 min. (**c**) Sample with 10 eq. TCEP and 50% MeCN. Product displays RT = 4.72 min and SM displays RT = 4.87 min.

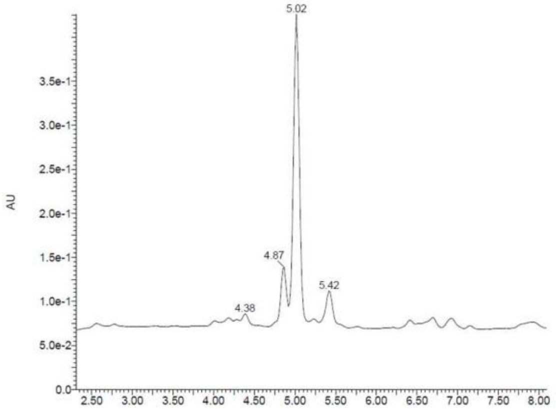

(**a**)

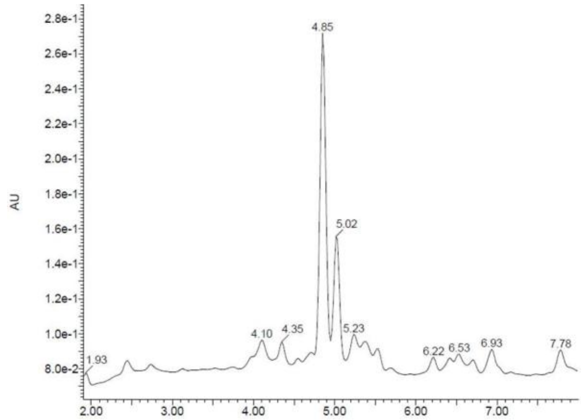

(**b**)

Figure S7. UV traces from LCMS optimizing pH in PB. Gradient: 20–40% B over 12 min. (**a**) Sample with pH = 6. Product displays RT = 4.87 min and SM displays RT = 5.02 min. (**b**) Sample with pH = 8. Product displays RT = 4.85 min and SM displays RT = 5.02 min.

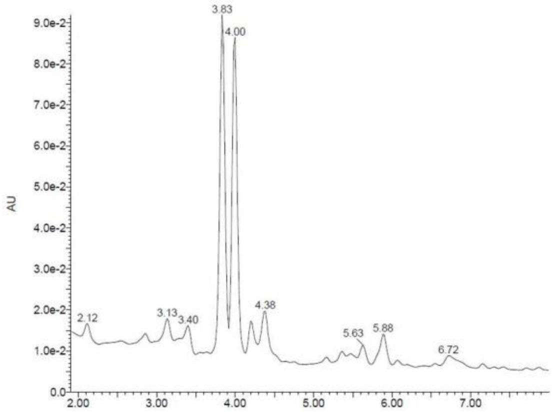

(**a**)

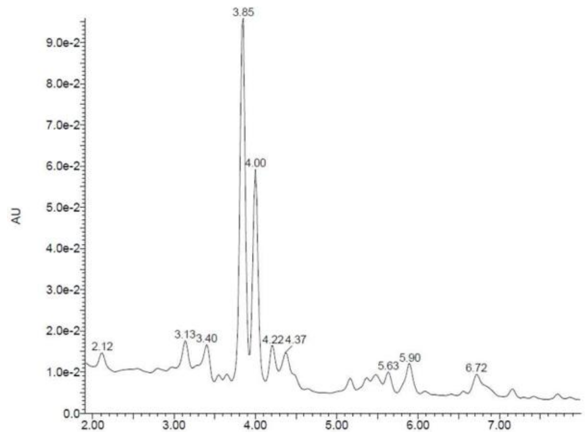

(**b**)

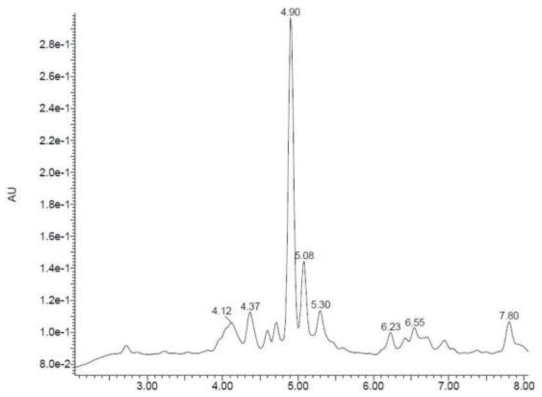

(**c**)

Figure S8. UV traces from LCMS optimizing pH in Tris buffer. Gradient: 20–40% B over 12 min. (**a**) Sample with pH = 7. Product displays RT = 3.83 min and SM displays RT = 4.00 min. (**b**) Sample with pH = 8. Product displays RT = 3.85 min and SM displays RT = 4.00 min. (c) Sample with pH = 9. Product displays RT = 4.90 min and SM displays RT = 5.08 min.

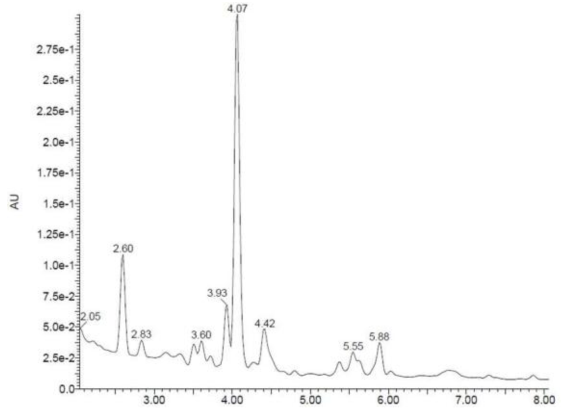

(**a**)

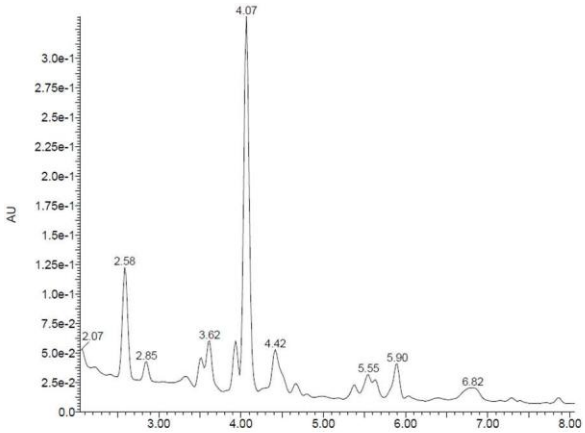

(**b**)

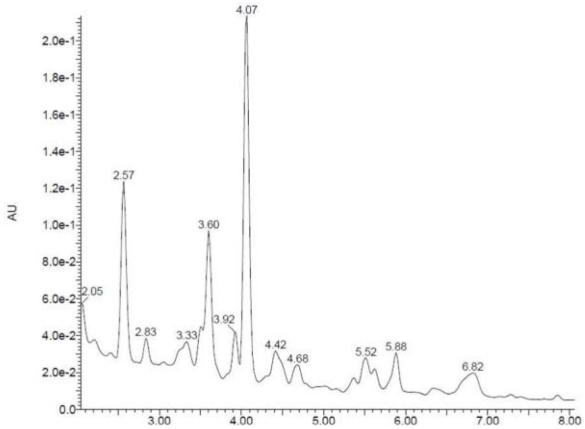

(**c**)

Figure S9. UV traces from LCMS optimizing pH in Tris buffer with 25% MeCN. Gradient: 20–40% B over 12 min. (**a**) Sample with pH = 7. Product displays RT = 3.93 min and SM displays RT = 4.07 min. (**b**) Sample with pH = 8. Product displays RT = 3.93 min and SM displays RT = 4.07 min. (c) Sample with pH = 9. Product displays RT = 4.92 min and SM displays RT = 4.07 min.

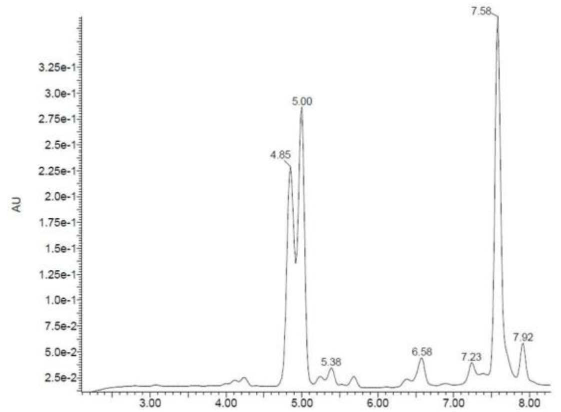

(**a**)

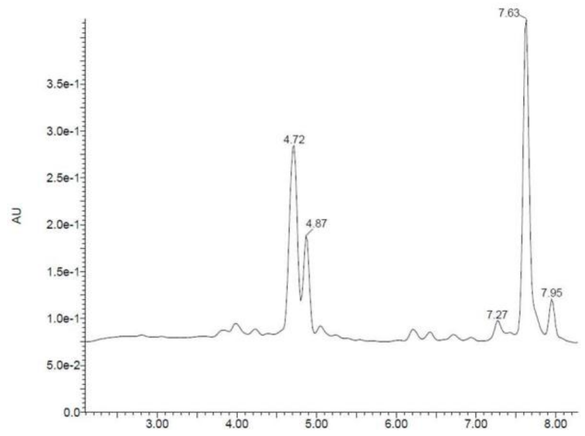

(**b**)

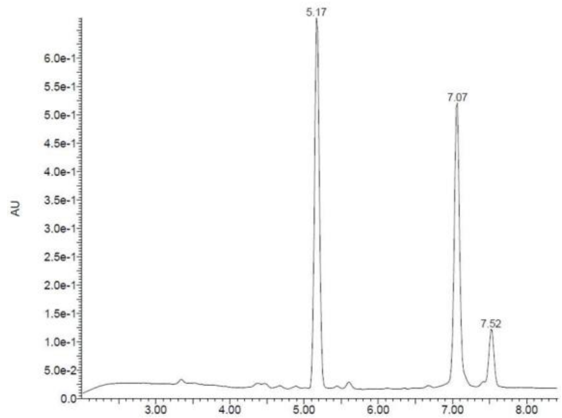

(**c**)

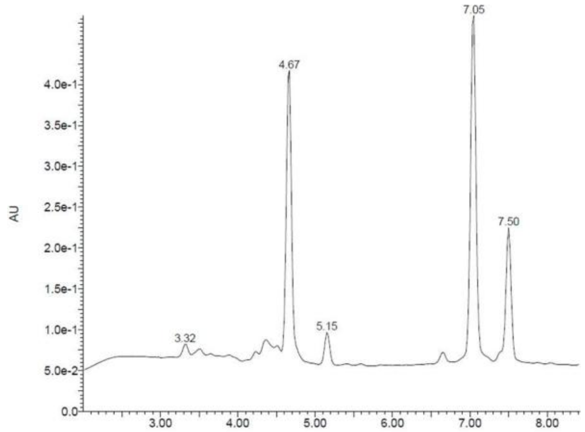

(**d**)

Figure S10. UV traces from LCMS for quantification using internal standard. (**a**) Sample with linear model peptide, not irradiated. SM displays RT = 4.85 and 5.00 min and internal standard displays RT = 7.58 min. Gradient: 20–40% B over 12 min. (**b**) Sample with linear model peptide, irradiated for 30 min. Products displays RT = 4.68 and 4.86 min, SM displays RT = 5.02 min, and internal standard displays RT = 7.63 min. Gradient: 20–40% B over 12 min. (**c**) Sample with head-to-tail cyclic model peptide, not irradiated. SM displays RT = 5.17 min and internal standard displays RT = 7.07 min. Gradient: 30–50% B over 12 min. (**d**) Sample with head-to-tail cyclic model peptide, irradiated for 30 min. Product displays RT = 4.67, SM displays RT = 5.15 min, and internal standard displays RT = 7.05 min. Gradient: 30–50% B over 12 min.

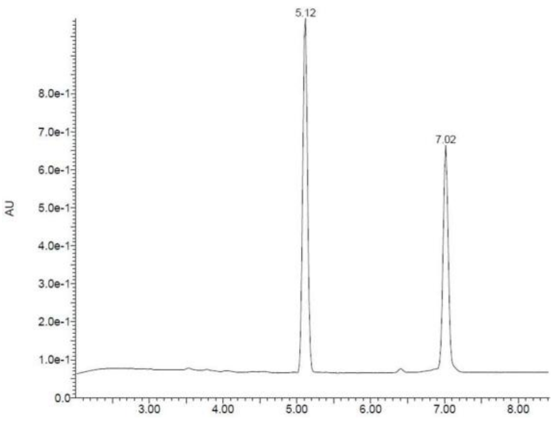

(**a**)

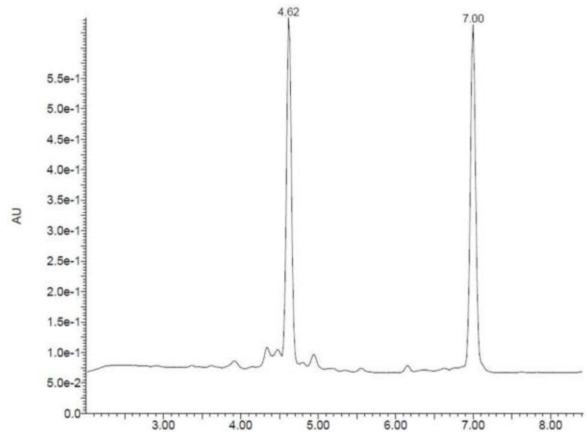

(**b**)

Figure S11. UV traces from LCMS of samples desulfurized with $NaBEt_4$. Gradient: 30–50% B over 12 min. (**a**) Control sample without $NaBEt_4$. SM displays RT = 5.12 min and internal standard displays RT = 7.02 min. (**b**) Sample with $NaBEt_4$. Product displays RT = 4.62 min and internal standard displays RT = 7.00 min.

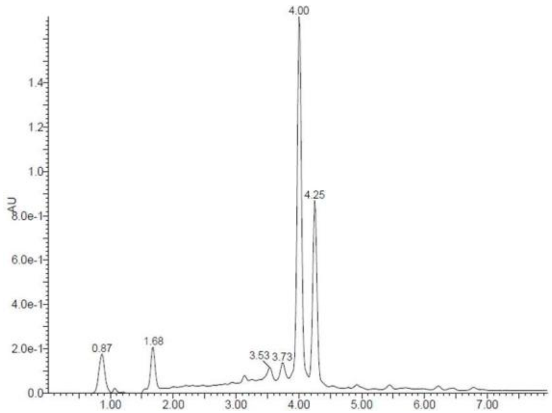

(**a**)

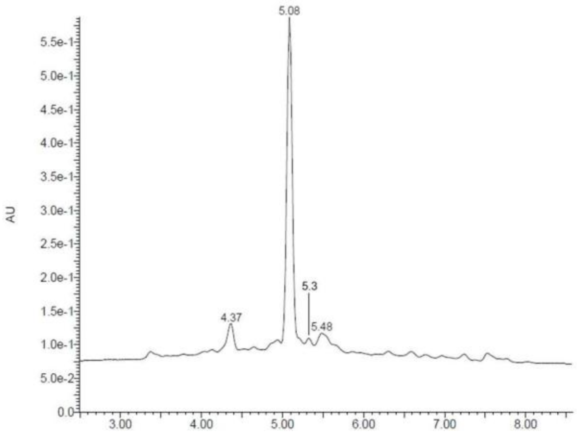

(**b**)

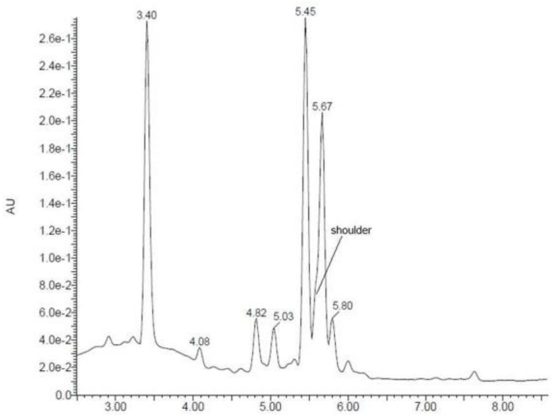

(**c**)

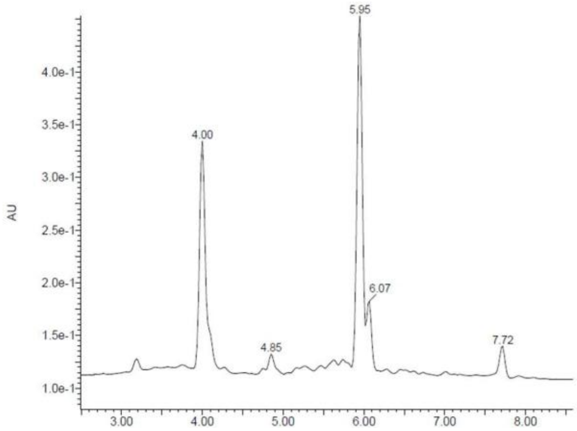

(**d**)

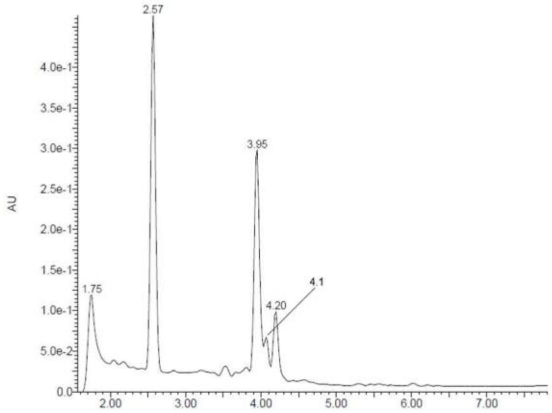

(**e**)

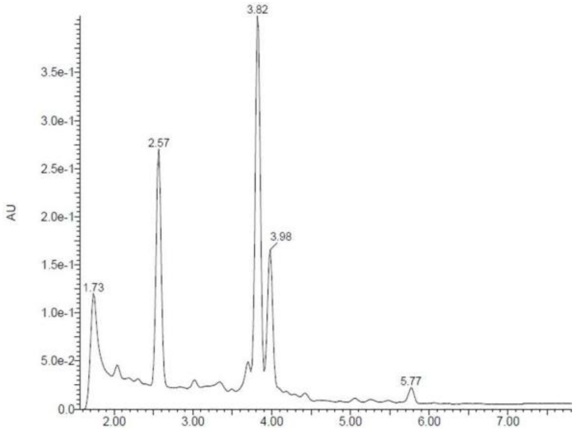

(**f**)

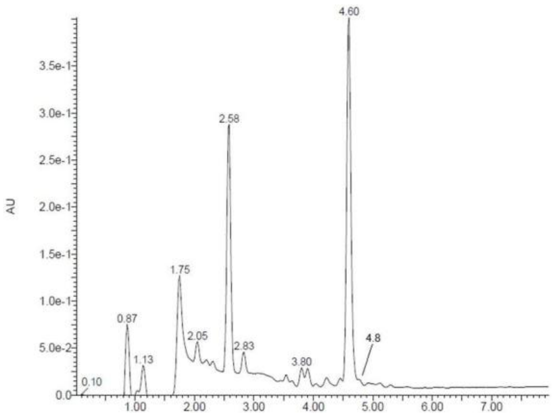

(**g**)

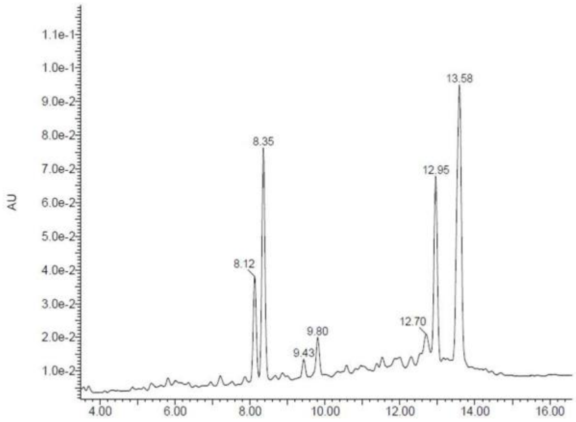

(**h**)

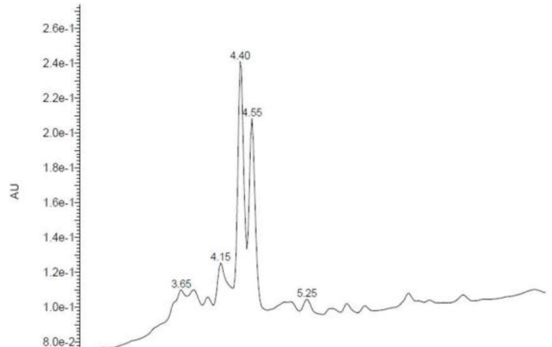

(**i**)

Figure S12. UV traces from LCMS of different peptide substrates after irradiation. (**a**) Peptide **3** displays RT = 4.00 min and SM displays RT = 4.25 SM. Peak with RT = 3.73 min is presumed to be an oxidized byproduct. Gradient: 25–45% B over 12 min. (**b**) Peptide **4** displays RT = 5.08 min and SM displays RT = 5.32 min. Gradient: 20–40% B over 12 min. (**c**) Peptide **5** displays RT = 5.45 min and SM displays RT = 5.67. Left shoulder of SM peak comprises desulfurized product of peptide with Asn or Asp deletion; SM with Asn/Asp deletion displays RT = 5.80 min. Gradient: 20–40% B over 12 min. (**d**) Peptide **6** displays RT = 5.95 min and SM displays RT = 6.05 min. Gradient: 10–30% B over 12 min. (**e**) Peptide **2*** fully desulfurized product displays RT = 3.95 min, partially desulfurized product displays RT = 4.07 min, and SM

displays RT = 4.20 min. Gradient: 20–40% B over 12 min. (**f**) Peptide **7** displays RT = 3.82 min and SM displays RT = 3.98 min. Gradient: 20–40% B over 12 min. (**g**) Peptide **8** displays RT = 4.60 min and SM displays RT = 4.77 min. Gradient: 20–40% B over 12 min. (**h**) Peptide **9** displays RT = 8.12 min and SM displays RT = 8.35 min. Peak with RT = 12.95 comprises SM with presumed ^{t}Bu protecting group. Peak with RT = 13.58 min comprises three analogues of a presumed dimer formed through the click reaction: desulfurized, unreacted, and partially desulfurized. Gradient: 20–40% B over 21 min. (**i**) Peptide **11** displays RT = 4.40 min and SM displays RT = 4.55 min. The presumed peptide with deprotected thiazolidine and desulfurization displays RT = 4.15 min. Gradient: 25–35% B over 12 min.

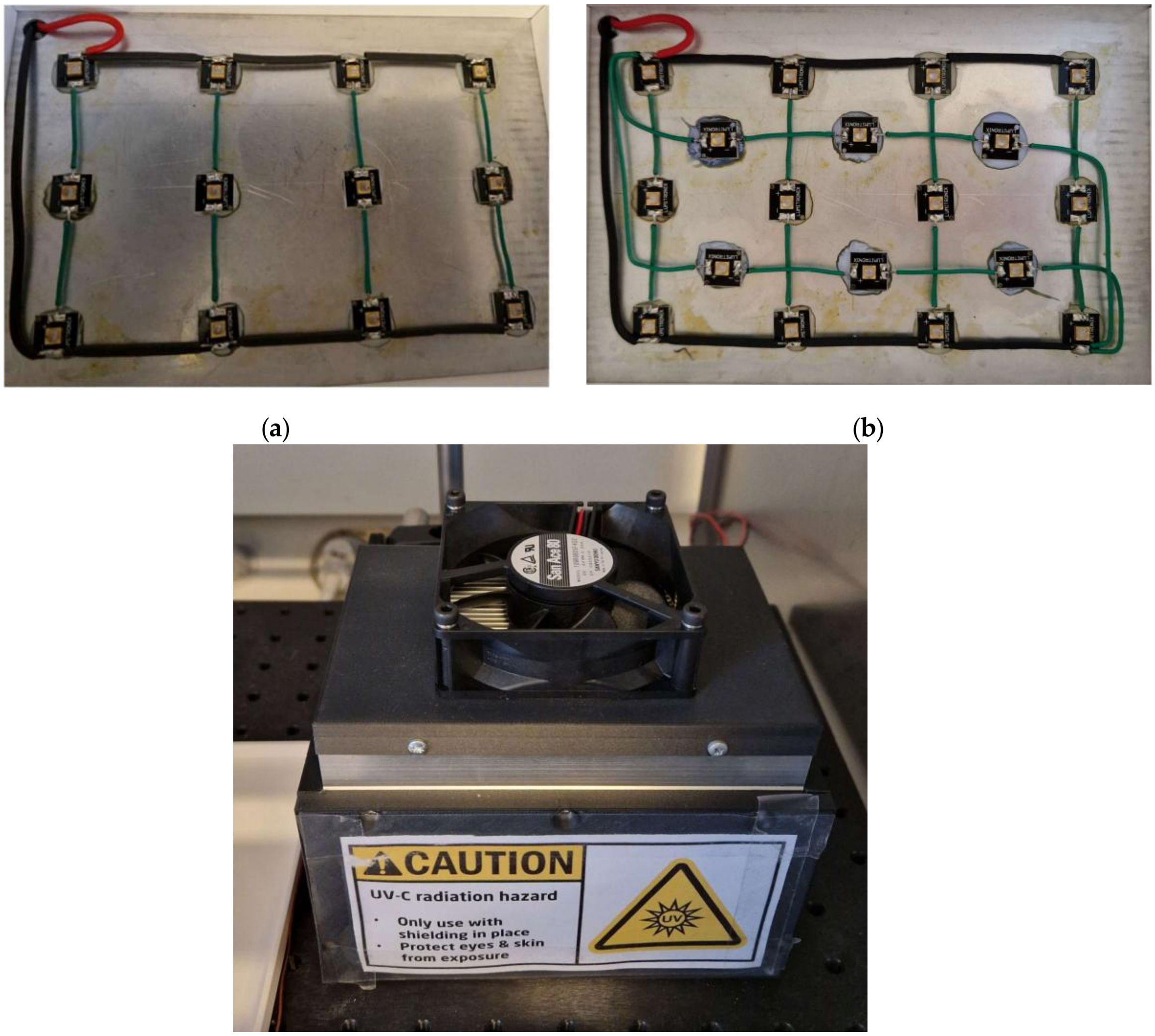

(**a**) (**b**) (**c**)

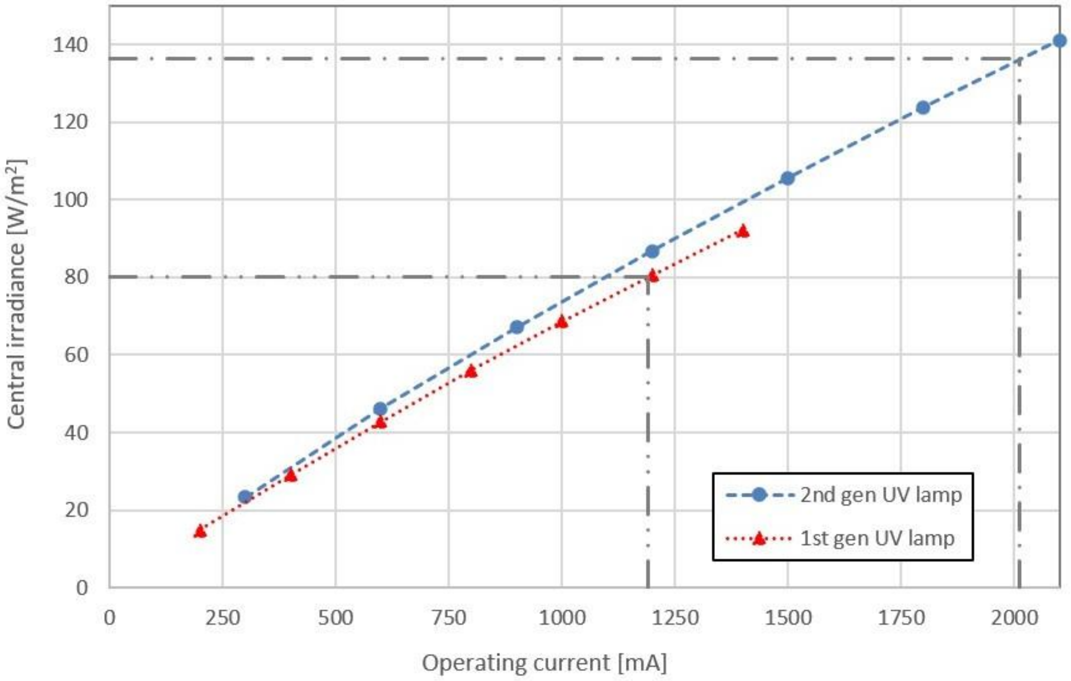

(**d**)

Figure S13. (**a**) LED array of first-generation lamp. (**b**) LED array of second-generation lamp. (**c**) Custom-made lamp system equipped with heat sinks and fan cooling. Inside, an LED array is mounted beneath the heat sink plate. The lamp sidewalls are covered on the inside with diffuse high reflectance PTFE sheets to enhance uniformity of the irradiance and further prevent UV light exposure outside. (**d**) Measured central irradiance as a function of operation current for the 1st and 2nd generation UV lamps at a distance of 82.5 mm from the LEDs, and the specific operating currents/central irradiances are marked.

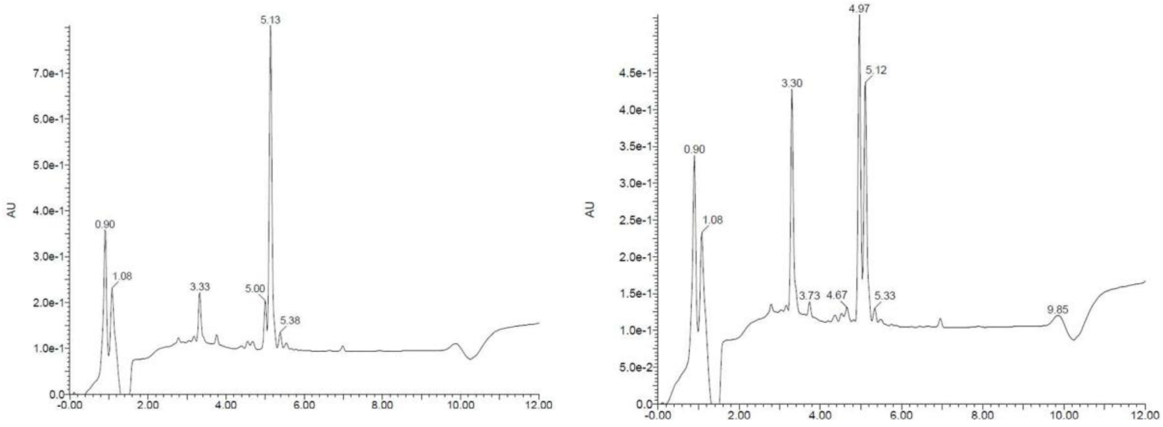

(**a**) (**b**)

Figure S14. Representative UV traces from LCMS of samples in 96-well plate irradiated with first-generation lamp at 35 mm distance from LED array for 45 min. Gradient: 20–40% B over 12 min. (**a**) Sample from well B10. Product displays RT = 5.00 min and SM displays RT = 5.13 min. (**b**) Sample from well D3. Product displays RT = 4.97 min and SM displays RT = 5.12 min.

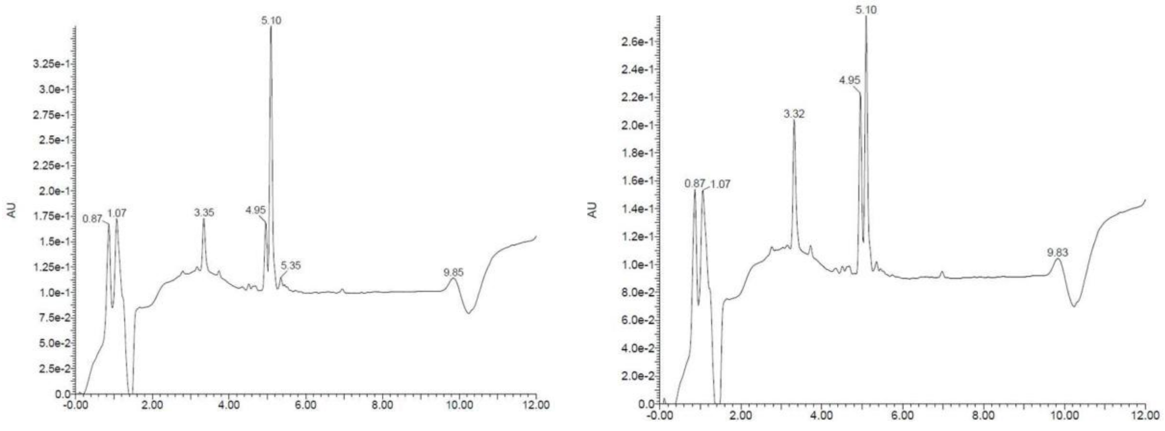

(**a**) (**b**)

Figure S15. Representative UV traces from LCMS of samples in 96-well plate irradiated with first-generation lamp at 42.5 mm for 45 min with the first-generation lamp. Gradient: 20–40% B over 12 min. (**a**) Sample from well B10. Product displays RT = 4.95 min and SM displays RT = 5.10 min. (**b**) Sample from well D3. Product displays RT = 4.95 min and SM displays RT = 5.10 min.

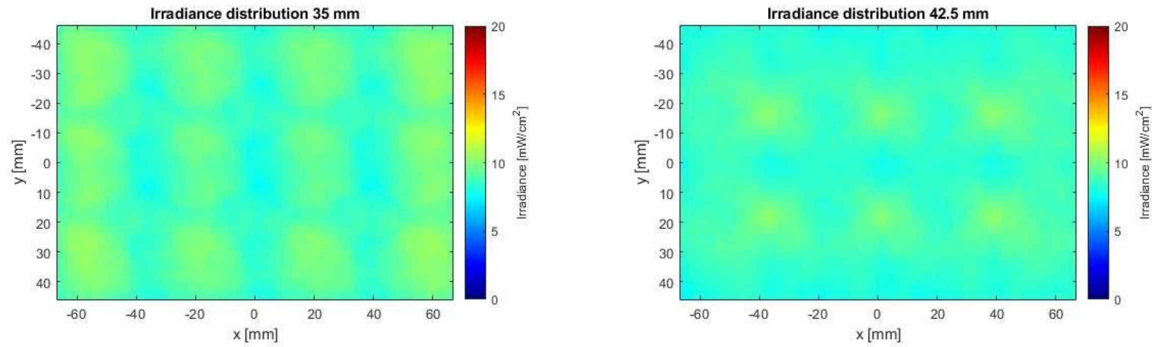

(**a**) (**b**)

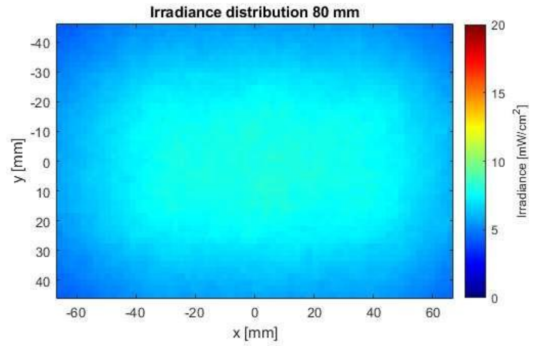

(**c**)

Figure S16. Simulated irradiance distribution (mW/cm^2) across detector dimensions at working distance of (**a**) 35, (**b**) 42.5, and (**c**) 80 mm from the first-generation lamp with an LED array comprising twelve aligned LEDs (3 × 4).

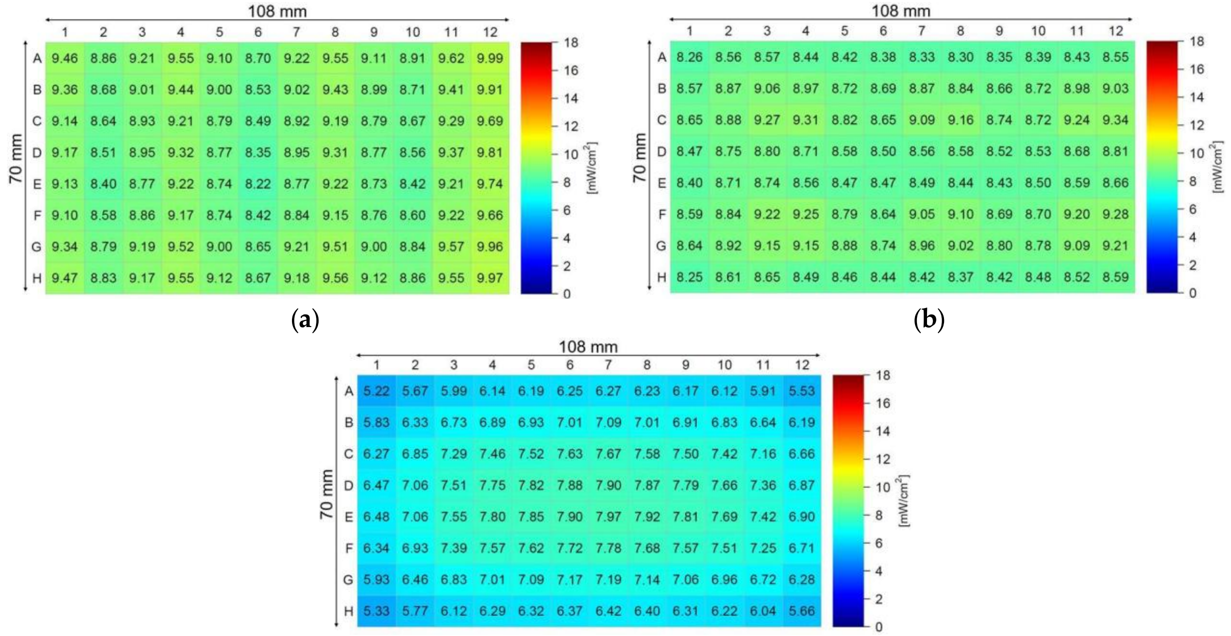

(c)

Figure S17. Average irradiance per well measured as irradiance distribution (mW/cm^2) across well-plate dimensions at working distance of (**a**) 35, (**b**) 42.5, and (**c**) 80 mm from the first-generation lamp with an LED array comprising twelve aligned LEDs (3 × 4).

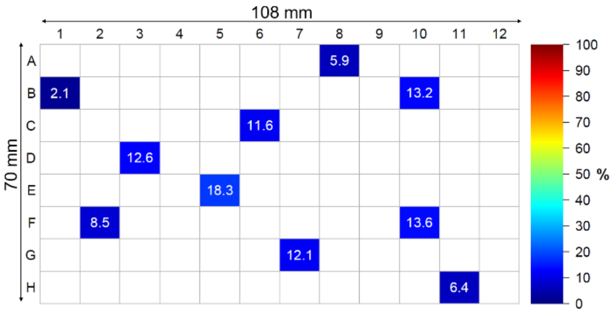

Figure S18. Heat map of product formation from ten randomly selected wells using the first-generation lamp at a distance of 80 mm from the LED array after 45 min irradiation.

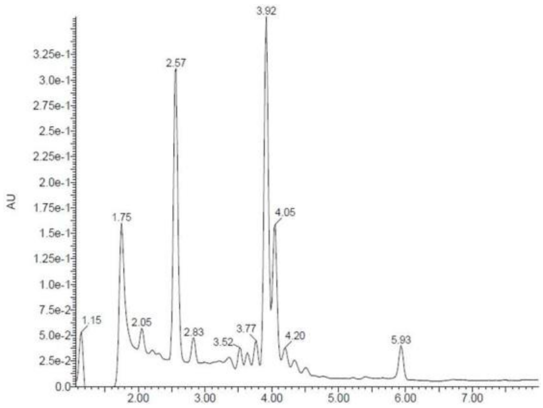

(**a**)

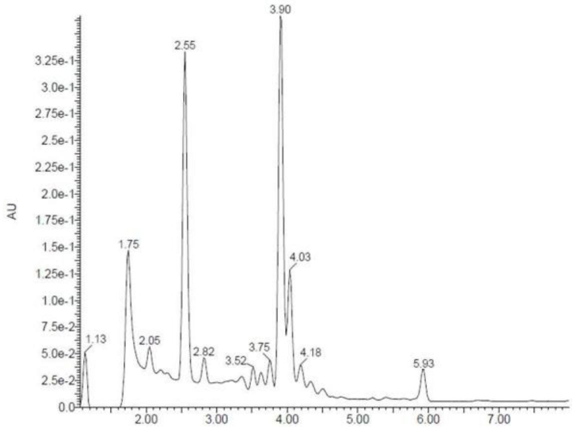

(**b**)

Figure S19. Representative UV traces from LCMS of samples in 96-well plate irradiated with the first-generation lamp at 80 mm for 180 min with the first-generation lamp. Gradient: 20–40% B over 12 min. (**a**) Sample from well B10. Product displays RT = 3.95 min and SM displays RT = 4.05 min. (**b**) Sample from well D3. Product displays RT = 3.90 min and SM displays RT = 4.03 min.

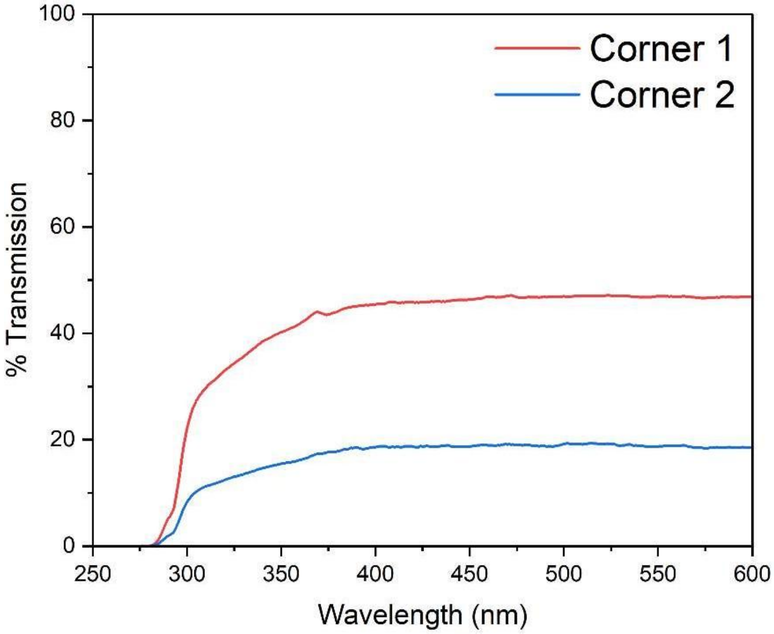

Figure S20. UV/vis transmission spectra of the polypropylene (PP) 96-well plate material. Measurements were taken from two separate pieces (corner 1 and 2) cut from the well plate.

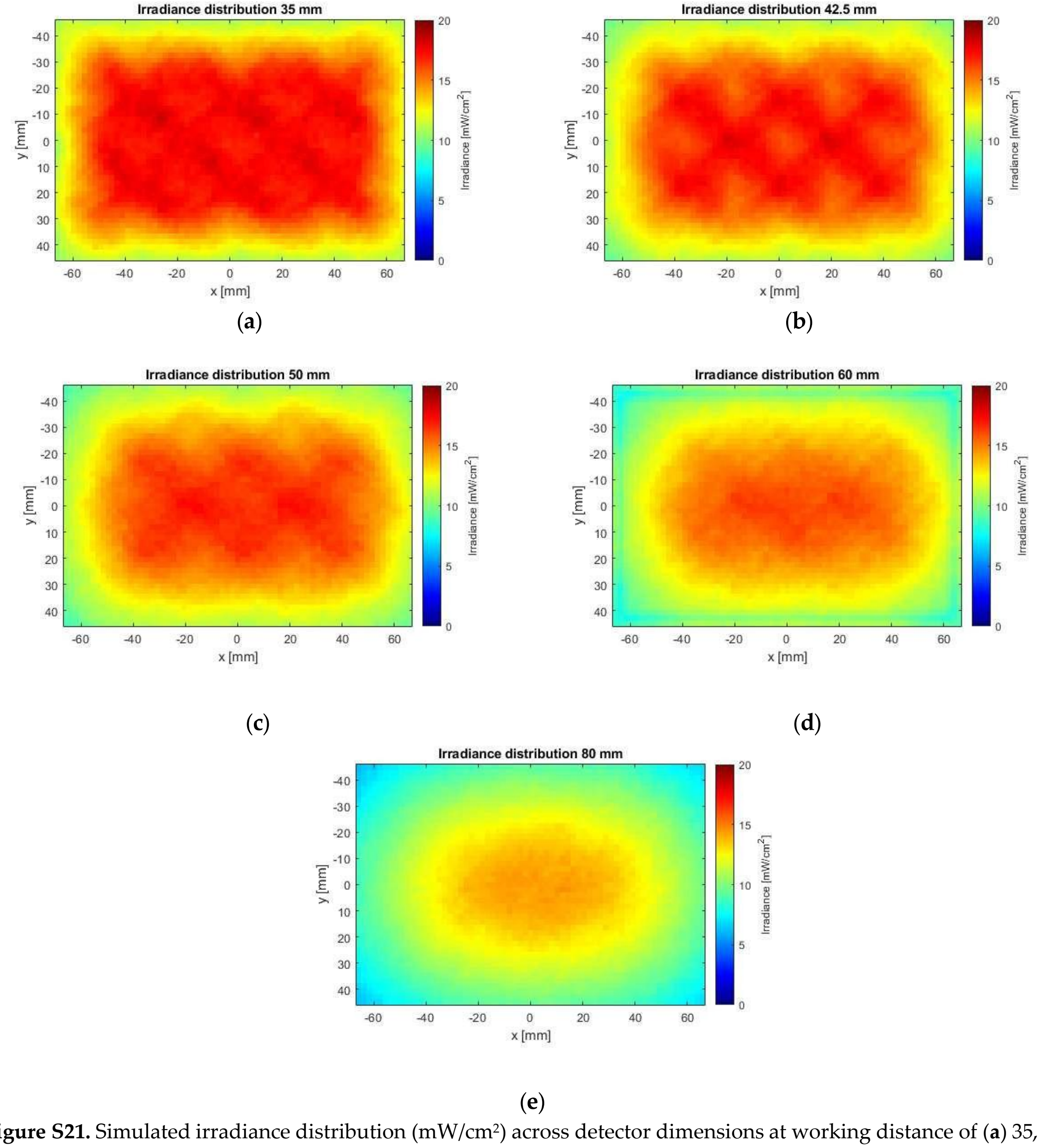

Figure S21. Simulated irradiance distribution (mW/cm²) across detector dimensions at working distance of (**a**) 35, (**b**) 42.5, (**c**) 50, (**d**) 60, and (**e**) 80 mm from an LED array comprising twelve central LEDs (3 × 4) plus 6 central LEDs (2 × 3).

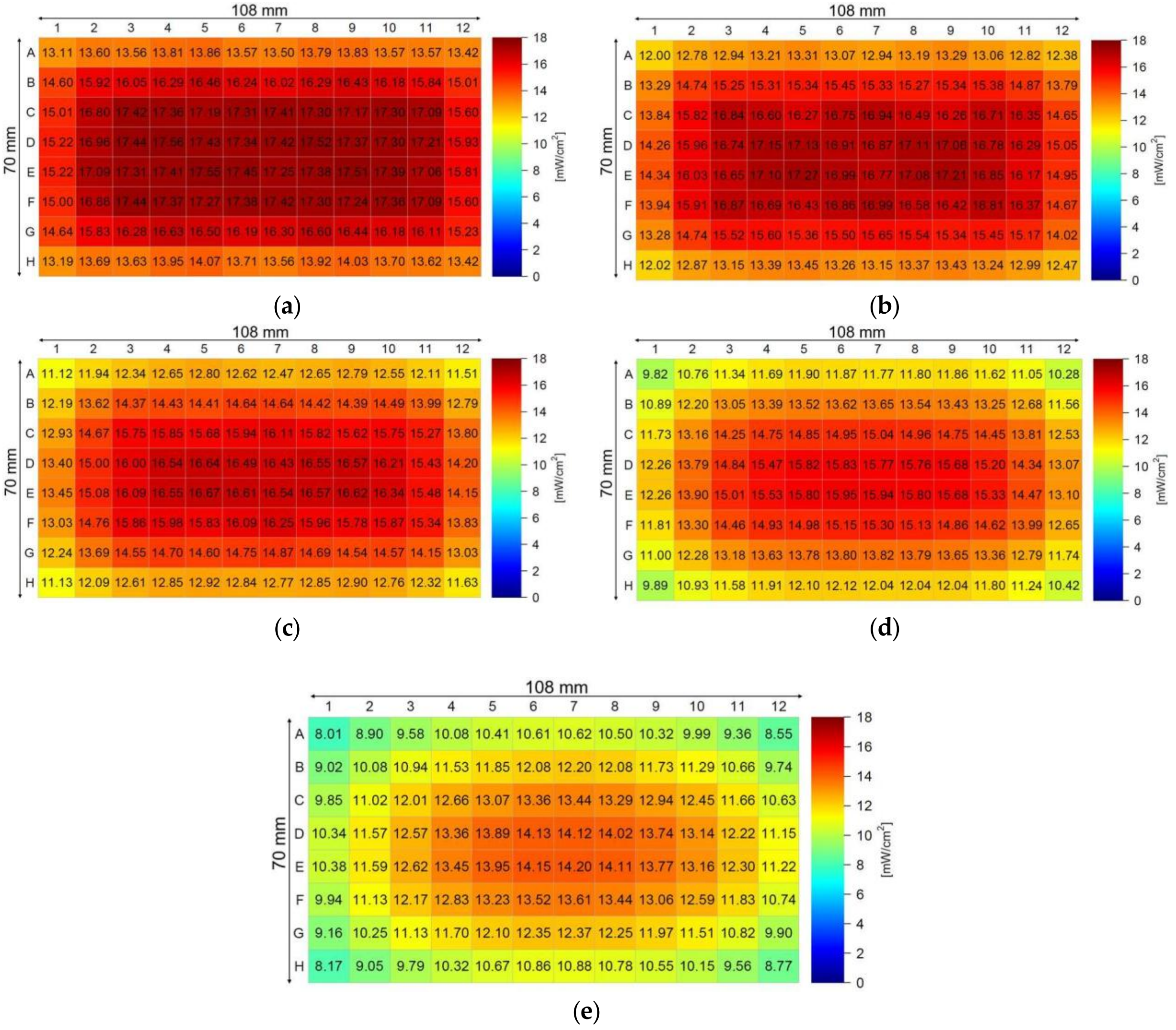

Figure S22. Average irradiance per well measured as irradiance distribution (mW/cm^2) across well-plate dimensions at working distance of (**a**) 35, (**b**) 42.5, (**c**) 50, (**d**) 60, and (**e**) 80 mm from an LED array comprising twelve LEDs (3 × 4) plus 6 central LEDs (2 × 3).

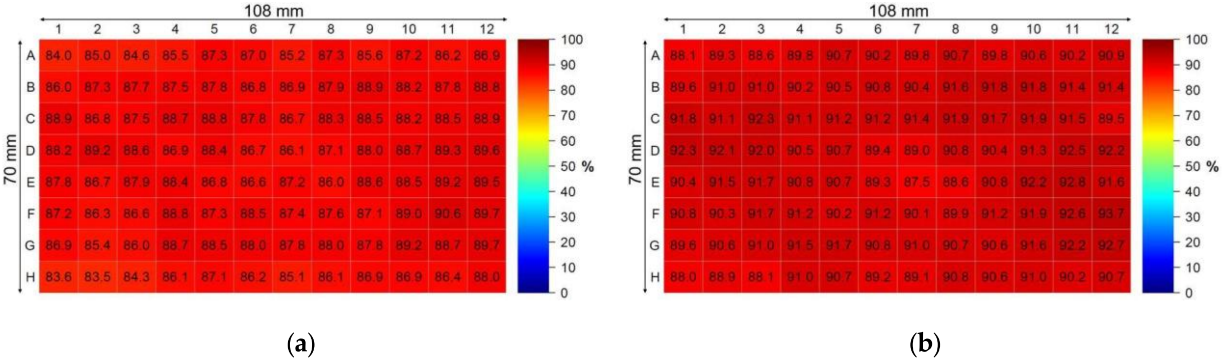

Figure S23. Heat maps of product formation irradiated with the second-generation lamp at a distance of 50 mm. (**a**) Product formation after 100 min, (**b**) and after 115 min.

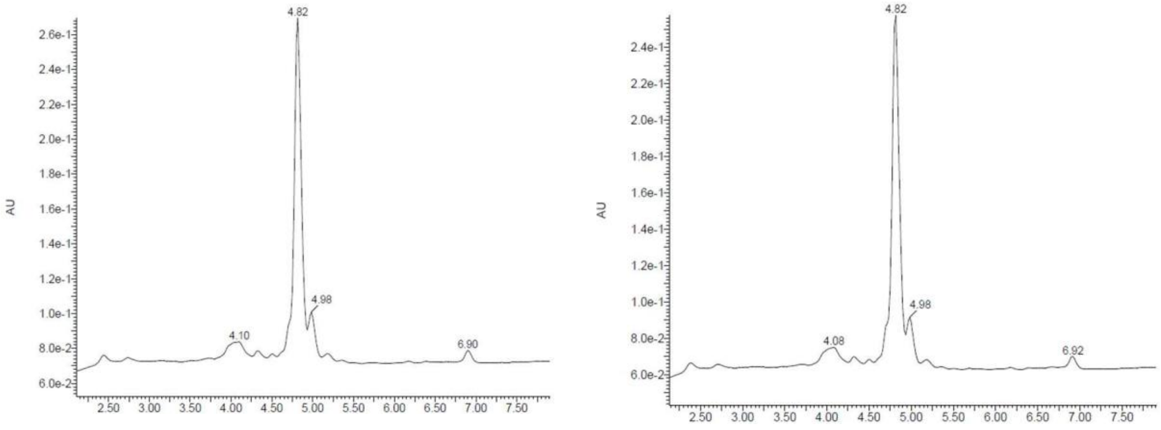

(**a**) (**b**)

Figure S24. Representative UV traces from LCMS of samples in 96-well plate irradiated with the second-generation lamp at 50 mm for 100 min with the second-generation lamp. Gradient: 20–40% B over 12 min. (**a**) Sample from well B10.

Product displays RT = 4.82 min and SM displays RT = 4.98 min. (**b**) Sample from well D3. Product displays RT = 4.82 min and SM displays RT = 4.98 min.

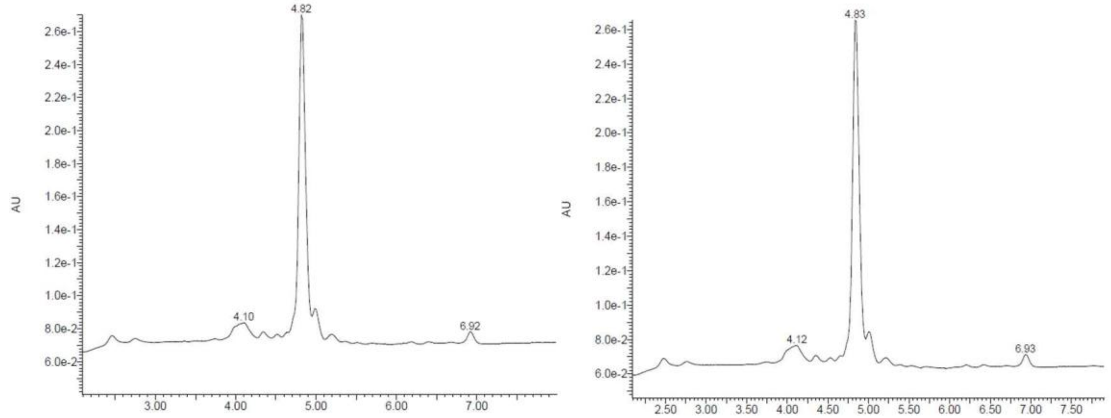

(**a**) (**b**)

Figure S25. Representative UV traces from LCMS of samples in 96-well plate irradiated with the second-generation lamp at 50 mm for 115 min with the second-generation lamp. Gradient: 20–40% B over 12 min. (**a**) Sample from well B10.

Product displays RT = 4.82 min and SM displays RT = 4.98 min. (**b**) Sample from well D3. Product displays RT = 4.83 min and SM displays RT = 4.98 min.

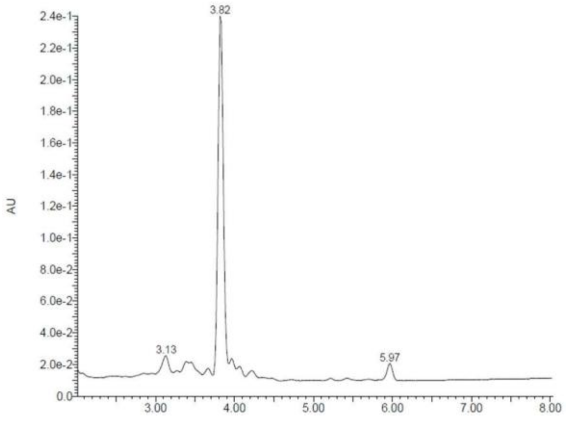

(**a**)

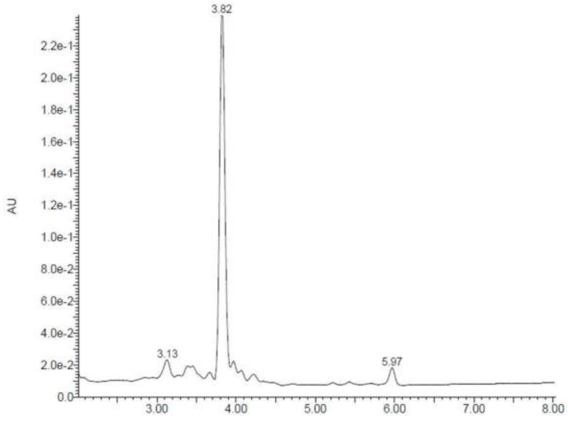

(**b**)

Figure S26. Representative UV traces from LCMS of samples in 96-well plate irradiated with the second-generation lamp at 50 mm for 130 min with the second-generation lamp. Gradient: 20–40% B over 12 min. (**a**) Sample from well B10.
Product displays RT = 3.82 min and SM displays RT = 3.98 min. (**b**) Sample from well D3. Product displays RT = 3.82 min and SM displays RT = 3.98 min.

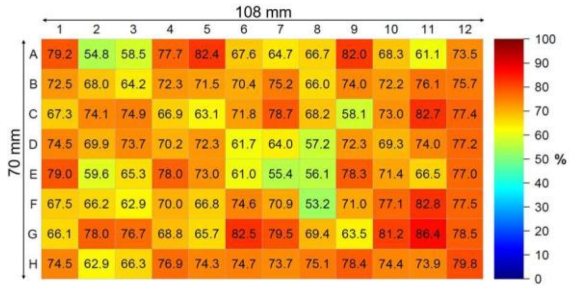

(**a**)

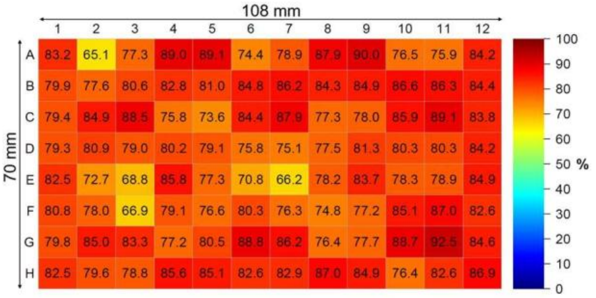

(**b**)

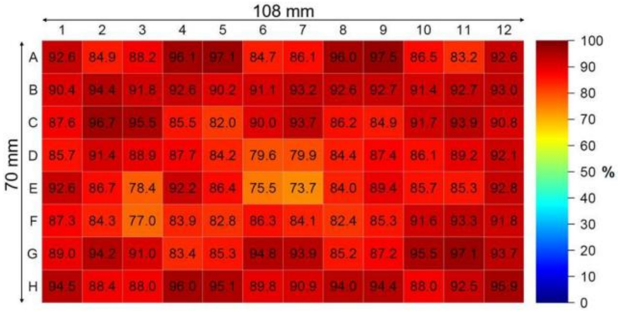

(**c**)

Figure S27. Heat maps of product formation using the second-generation lamp at 35 mm. (**a**) Product formation after 45 min,
(**b**) after 60 min, and (**c**) after 75 min.

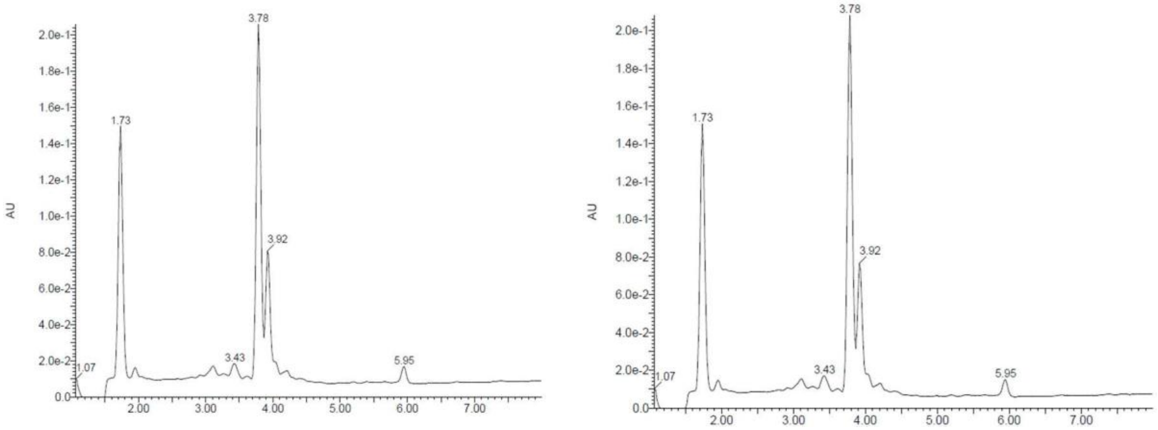

(a) (b)

Figure S28. Representative UV traces from LCMS of samples in 96-well plate irradiated with the second-generation lamp at 35 mm for 45 min with the second-generation lamp. Gradient: 20–40% B over 12 min. (**a**) Sample from well B10.
Product displays RT = 3.78 min and SM displays RT = 3.92 min. (**b**) Sample from well D3. Product displays RT = 3.78 min and SM displays RT = 3.92 min.

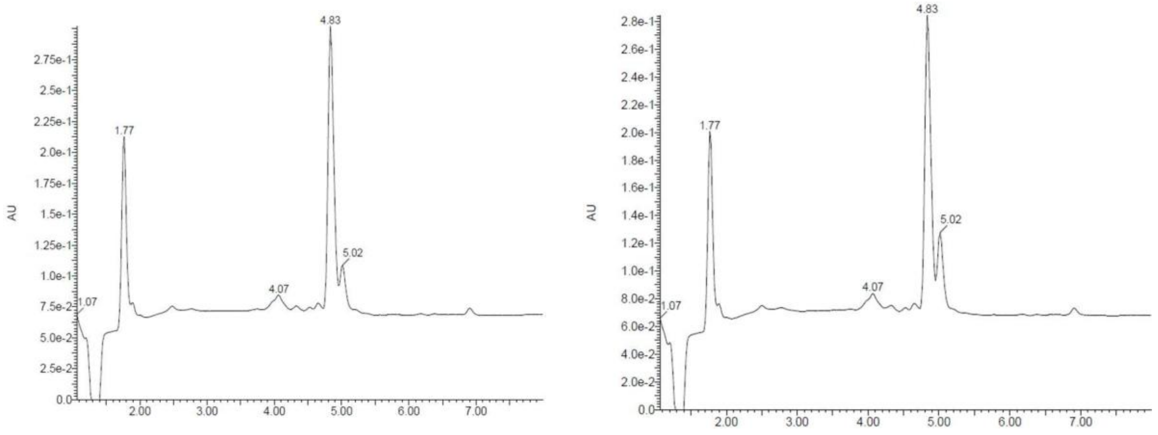

(a) (b)

Figure S29. Representative UV traces from LCMS of samples in 96-well plate irradiated with the second-generation lamp at 35 mm for 60 min with the second-generation lamp. Gradient: 20–40% B over 12 min. (**a**) Sample from well B10.
Product displays RT = 4.83 min and SM displays RT = 5.02 min. (**b**) Sample from well D3. Product displays RT = 4.83 min and SM displays RT = 5.02 min.

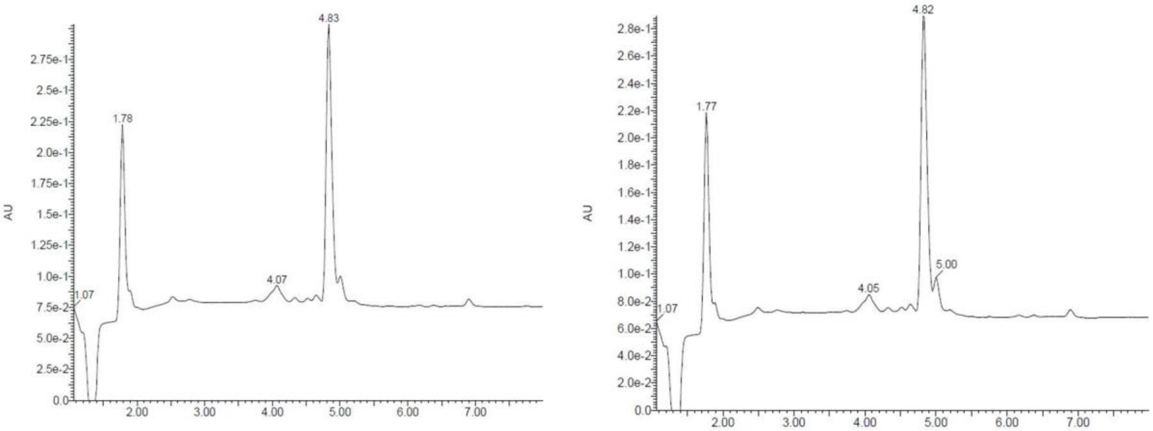

(**a**) (**b**)

Figure S30. Representative UV traces from LCMS of samples in 96-well plate irradiated with the second-generation lamp at 35 mm for 75 min with the second-generation lamp. Gradient: 20–40% B over 12 min. (**a**) Sample from well B10.

Product displays RT = 4.83 min and SM displays RT = 5.01 min. (**b**) Sample from well D3. Product displays RT = 4.82 min and SM displays RT = 5.00 min.

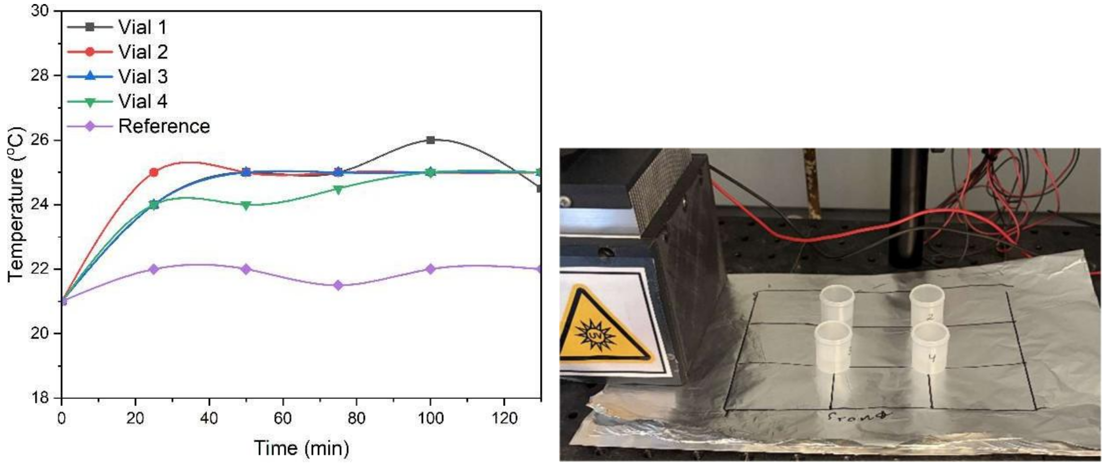

(a) **(b)**

Figure S31. Temperature monitoring during prolonged irradiation by the second-generation lamp. (**a**) Temperature profile of four irradiated water samples and a non-irradiated water reference over a 130 min period. (**b**) Experimental setup showing even placement of the four plastic vials.

Table S1 NMR chemical shift assignments of the resonances for model peptide **1**.

	NH/ppm	H_α/ppm	H_β/ppm	H_γ/ppm	H_δ/ppm	C_α/ppm	C_β/ppm	C_γ/ppm	C_δ/ppm	^{15}NH/ppm
Leu	-	3,76	1,56/1,51	1,65	0,89/0,88	50,7	40,1	23,4	22,6/21,7	-
Asn*	8,72	4,60	2,64/2,48	-	-	49,5	36,8	-	-	120,8
Asp	8,40	4,55	2,69/2,50	-	-	49,6	35,8	-	-	118,2
Ser	7,85	4,24	3,63/3,60	-	-	55,4#	61,4	-	-	112,4
Ala	7,97	4,27	1,24	-	-	48,3	17,6	-	-	121,7
Cys	7,92	4,34	2,76/2,68	-	-	55,0	26,0	-	-	114,3
Ala	8,05	4,24	1,21	-	-	48,3#	17,9	-	-	122,0
Leu	7,95	4,30	1,46	1,59	0,87/0,82	50,1	40,4	24,1	22,9/21,6	117,7
Val	7,70	4,13	1,98	0,83/0,81	-	57,5	30,4	19,1/18,0	-	114,7
Glu	8,01	4,24	1,90/1,75	2,22	-	51,9#	27,0	30,0	-	119,8
Glu	7,82	4,16	1,90/1,74	2,20	-	51,7	27,3	30,0	-	117,5
NH_2¤	7,30/7,08	-	-	-	-	-	-	-	-	104,9

NMR assignments for model compound **1**.

* Asn NH_2 δ_H 7,06/7,54 ppm; δ_N 110,8 ppm.

# The assignments may be interchanged.

¤ This is an NH_2 positioned on the C-terminal carbonyl.

Another compound is present in the spectra. LCMS analysis showed that this was most likely the disulfide dimer, formed over time when the sample was standing dissolved in DMSO-*d6* [38].

Figure S32. 1D ^{1}H NMR spectrum of model peptide **1** (800 MHz, DMSO-*d6*).

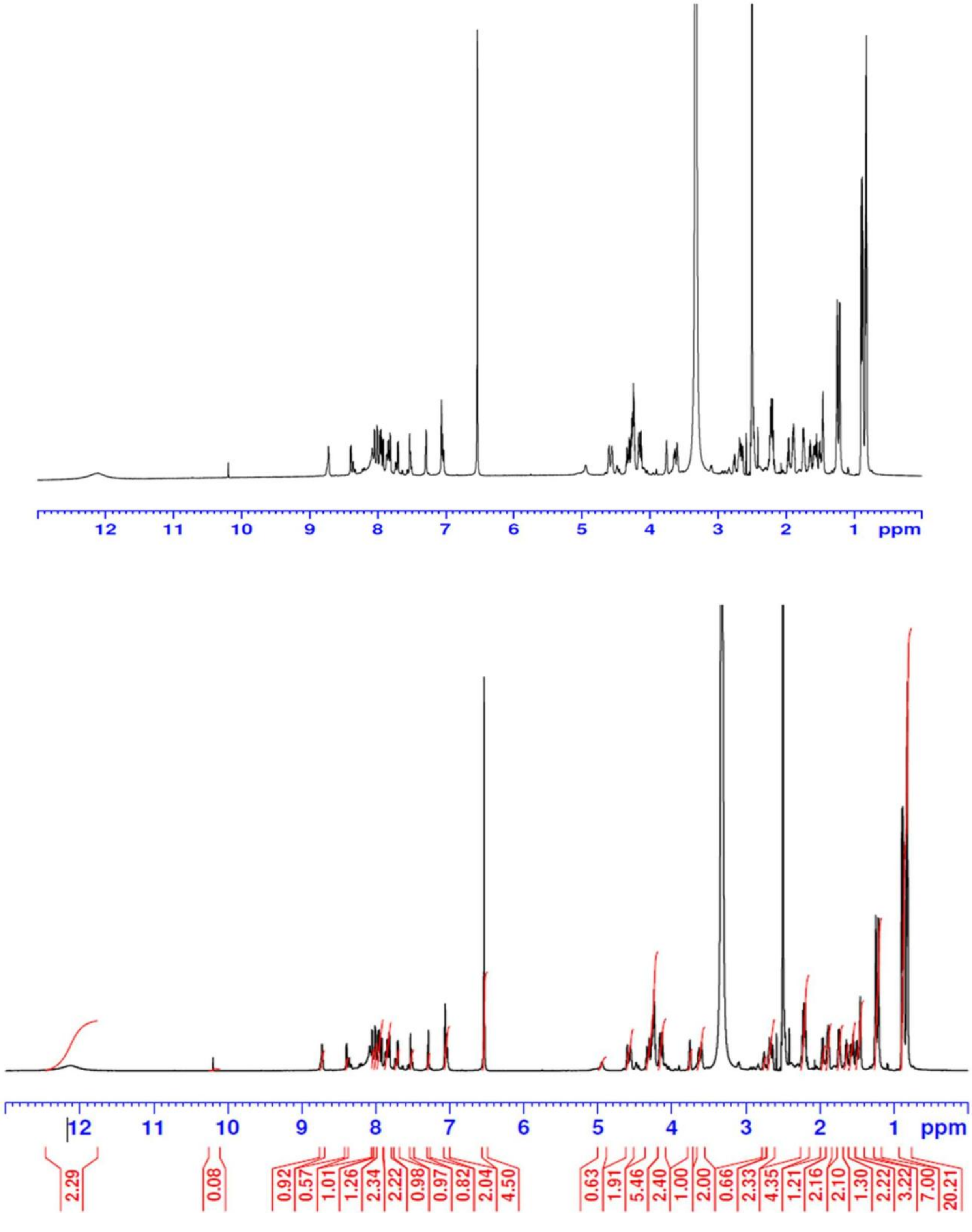

Figure S33. 2D ^1H-^1H DQF-COSY spectrum of model peptide **1** (800 MHz, DMSO-*d6*).

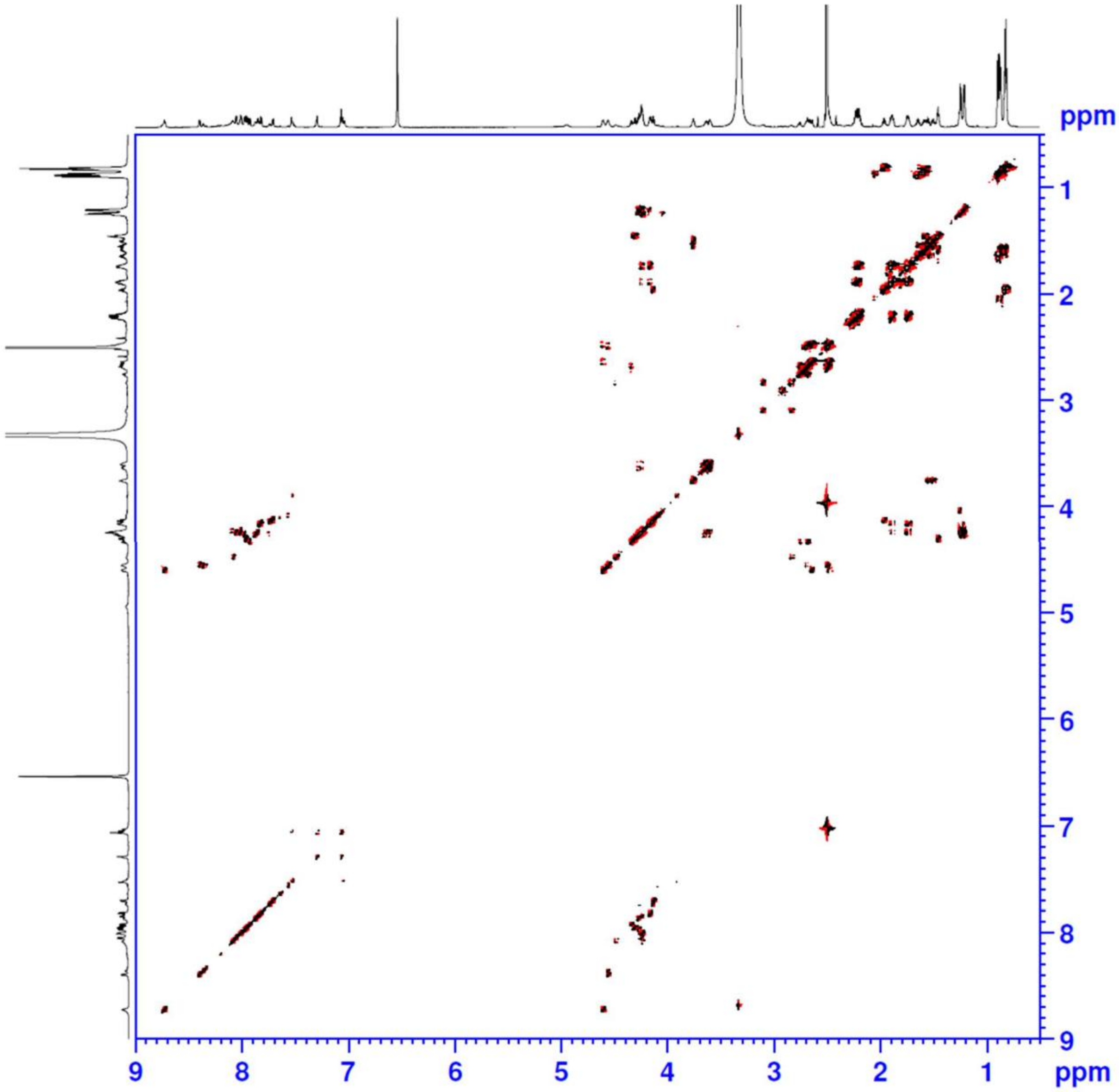

Figure S34. 2D ^{1}H-^{1}H TOCSY spectrum of model peptide **1** (800 MHz, DMSO-*d6*).

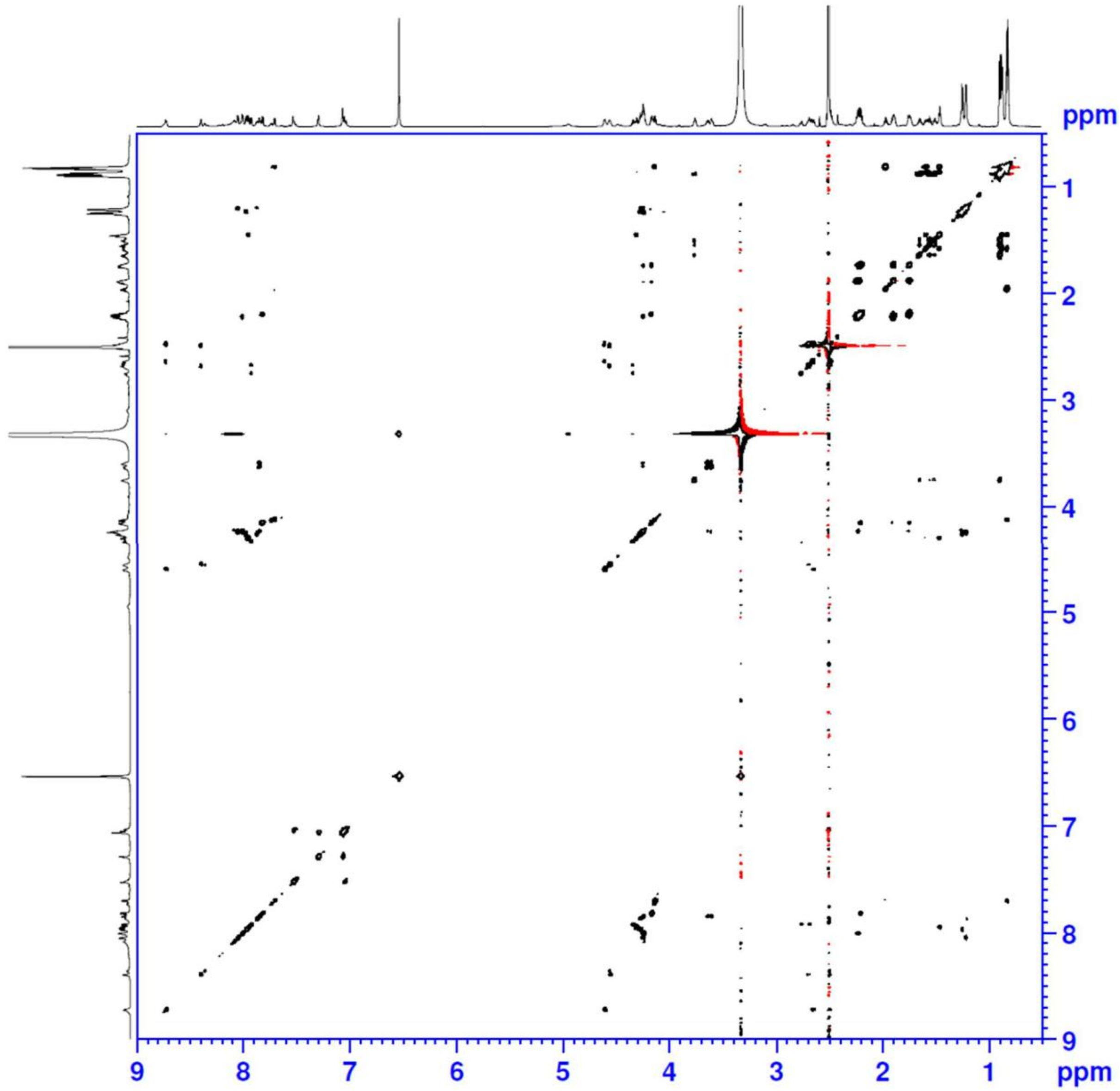

Figure S35. 2D ^1H-^1H NOESY spectrum of model peptide **1** (800 MHz, DMSO-*d6*).

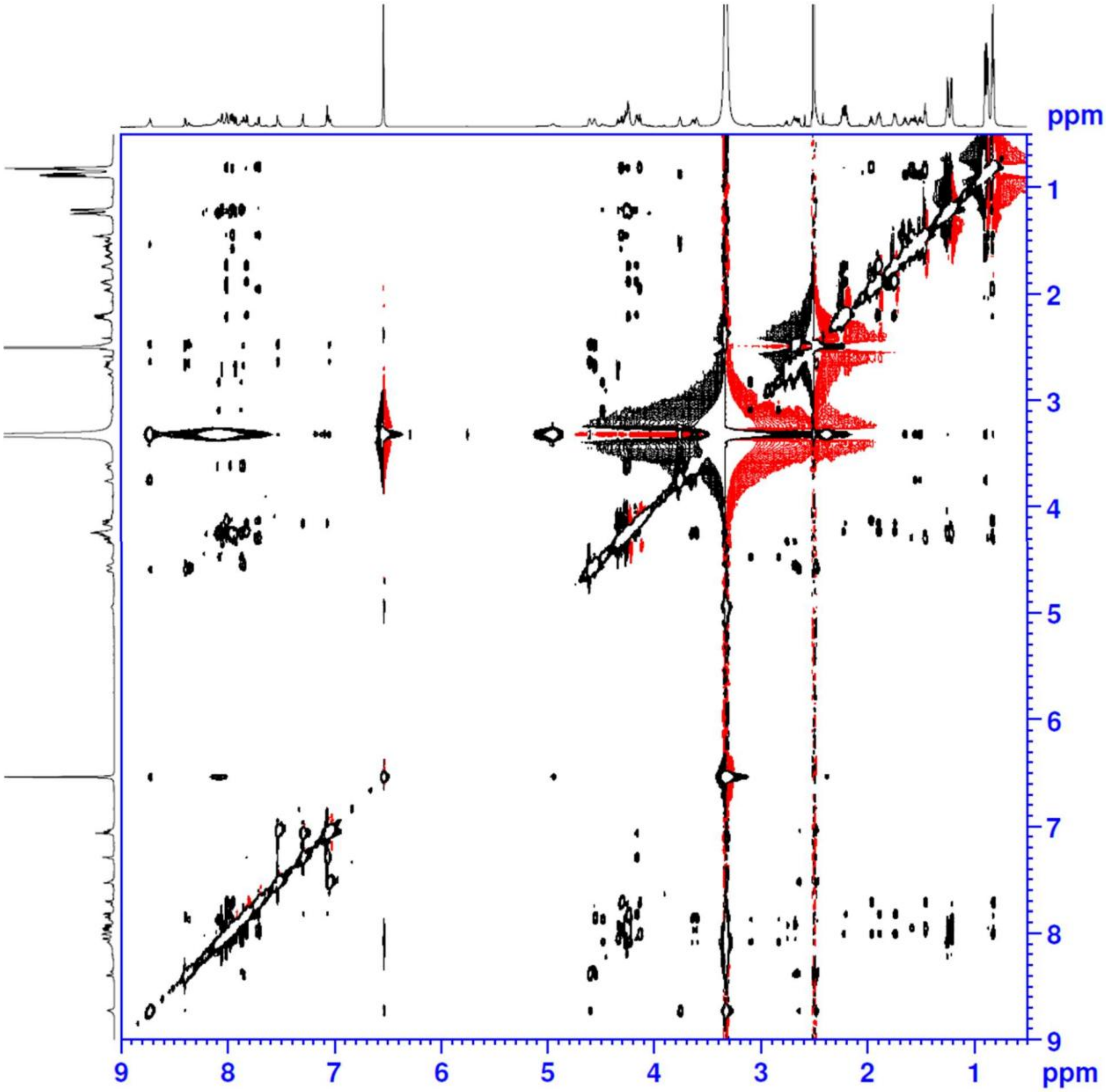

Figure S36. 2D ^{1}H-^{13}C edHSQC spectrum of model peptide **1** (800 MHz, DMSO-*d6*).

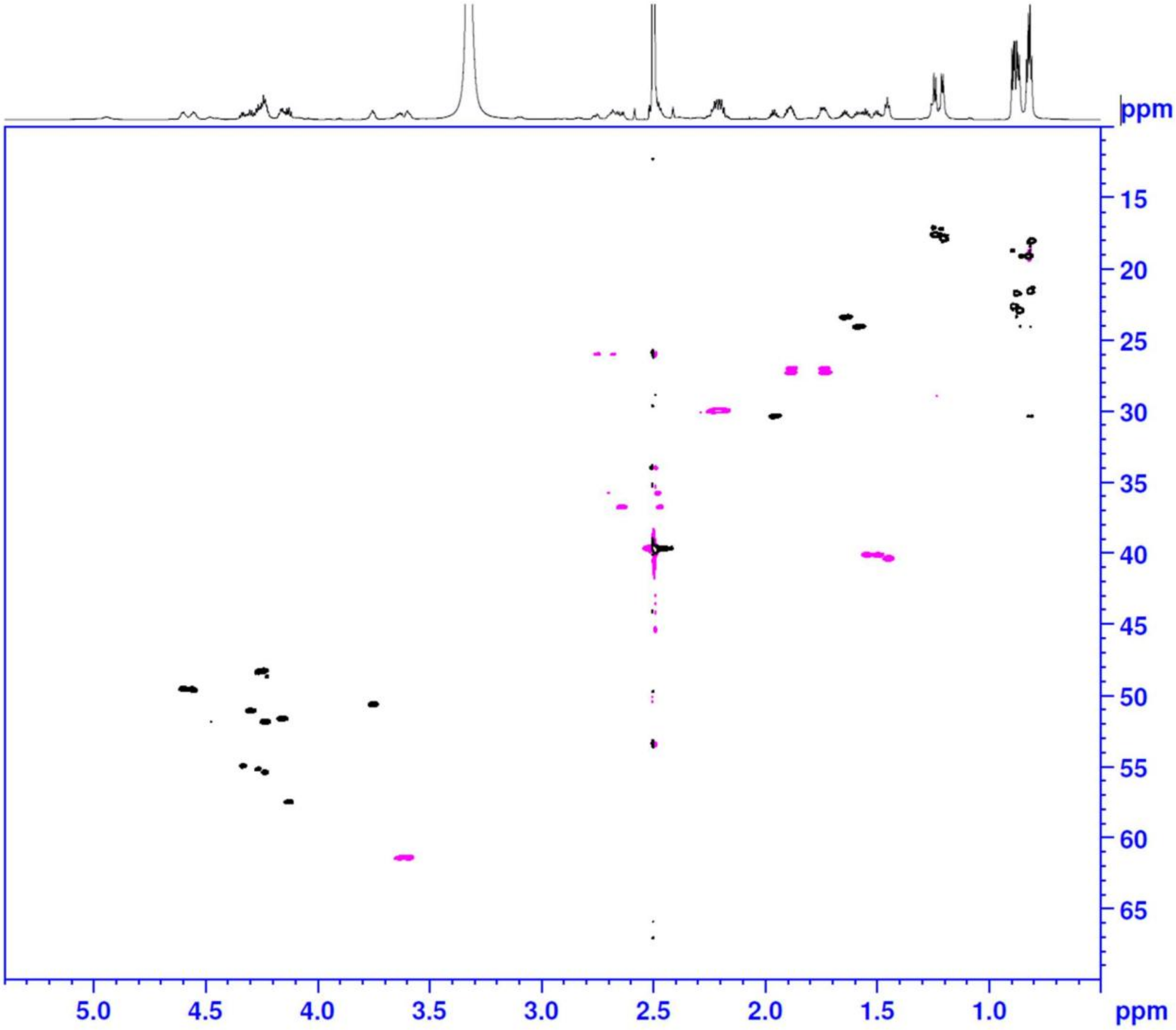

Figure S37. 2D ^1H-^{15}N HSQC spectrum of model peptide **1** (800 MHz, DMSO-*d6*).

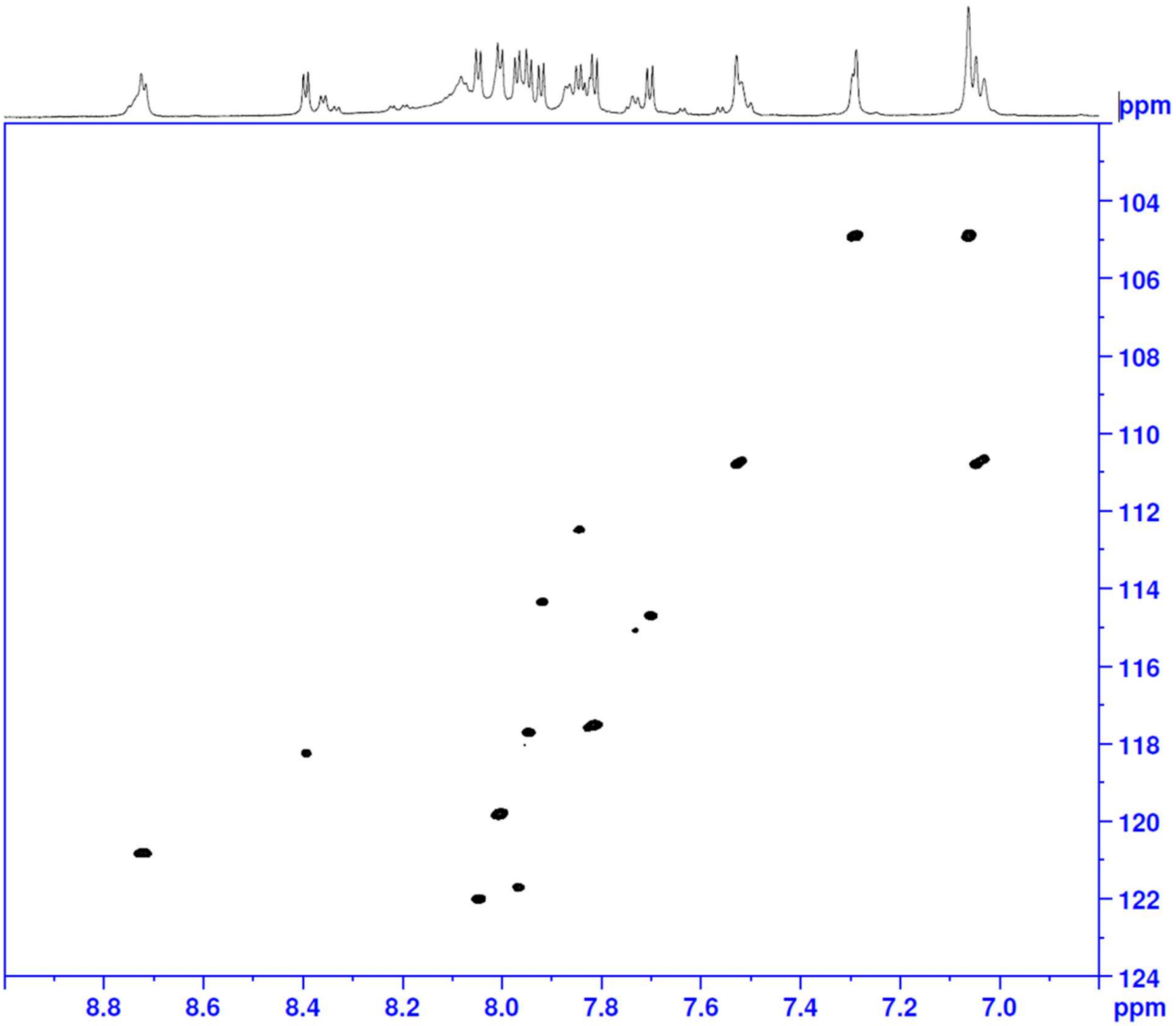

Table S2 NMR chemical shift assignments of the resonances for product **2**.

	NH/ppm	Hα/ppm	Hβ/ppm	Hy/ppm	Hδ/ppm	Cα/ppm	Cβ/ppm	Cy/ppm	Cδ/ppm	^{15}NH/ppm
Leu	-	3,76	1,56/1,51	1,65	0,89	50,5	40,7	24,0	22,3/23,2-	
Asn*	8,71	4,60	2,64/2,49	-	-	49,4	37,3	-	-	120,8
Asp	8,40	4,55	2,69/2,49	-	-	49,6	36,3	-	-	118,3
Ser	7,85	4,23	3,64/3,59	-	-	55,4	61,9	-	-	112,7
Ala	7,89	4,19	1,21	-	-	48,3	18,1	-	-	118,8
Ala	7,96	~4,21	1,24	-	-	48,2	18,0	-	-	121,8
Ala	7,85	4,21	1,22	-	-	48,3	18,3	-	-	118,7
Leu	7,83	4,27	1,48	1,59	0,88/0,83	51,0	40,9	24,6	22,1/23,5	117,1
Val	7,67	4,10	1,98	0,83	-	57,6	30,8	19,6/18,6	-	114,5
Glu	7,96	~4,23	1,90/1,75	2,21/2,24	-	51,8	27,5	30,6	-	119,5
Glu	7,78	4,15	1,90/1,74	2,21/2,24	-	51,5	27,8	30,6	-	117,3
NH_2¤	7,26/7,07	-	-	-	-	-	-	-	-	104,7

NMR assignments for product **2**.

* Asn NH_2 δ_H 7,54/7,07 ppm; δ_N 110,8 ppm.

¤ This is an NH_2 positioned on the C-terminal carbonyl.

Figure S38. 1D ^{1}H NMR spectrum of product **2** (800 MHz, DMSO-*d6*).

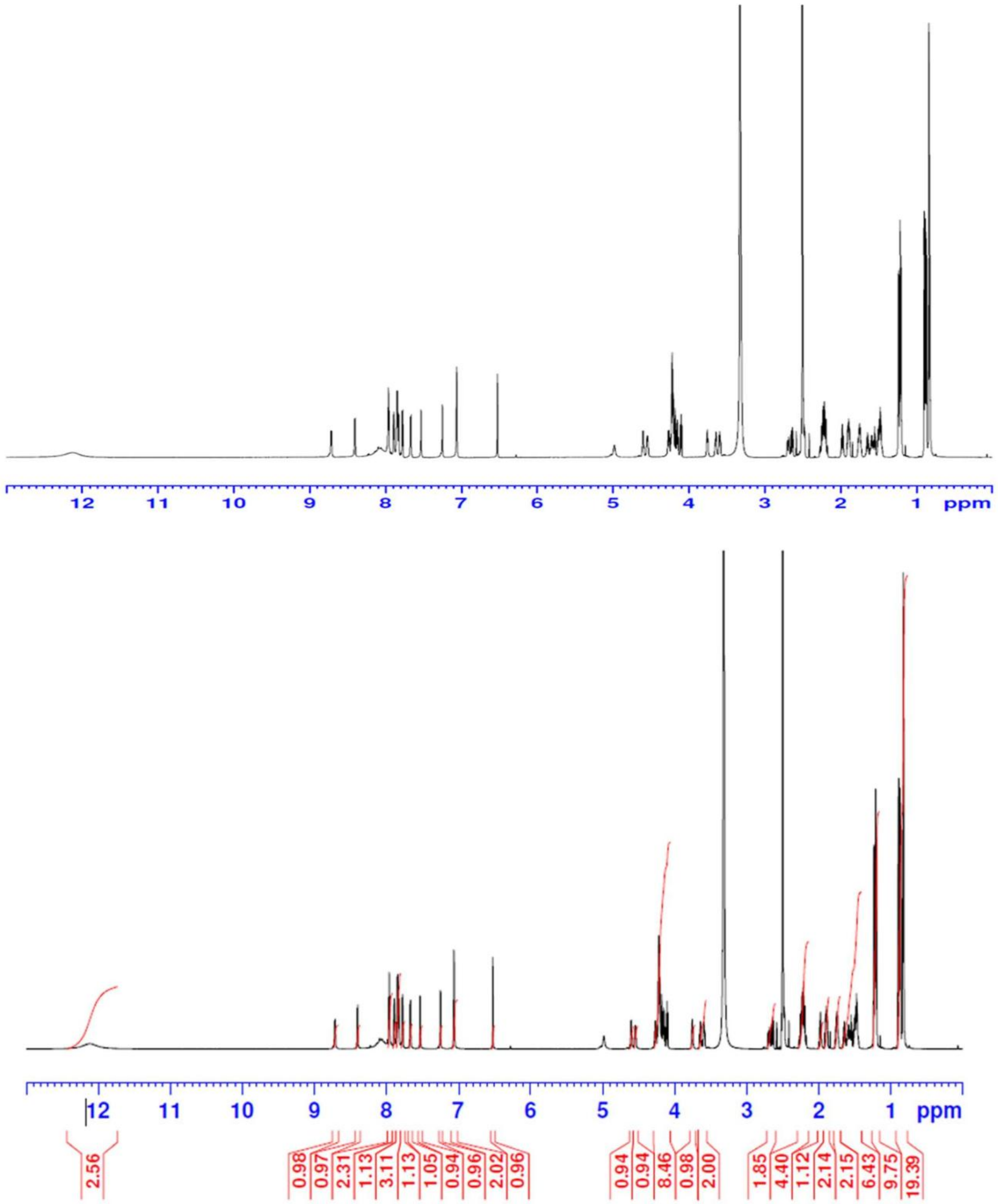

Figure S39. 2D ^1H-^1H DQF-COSY spectrum of product **2** (800 MHz, DMSO-*d6*).

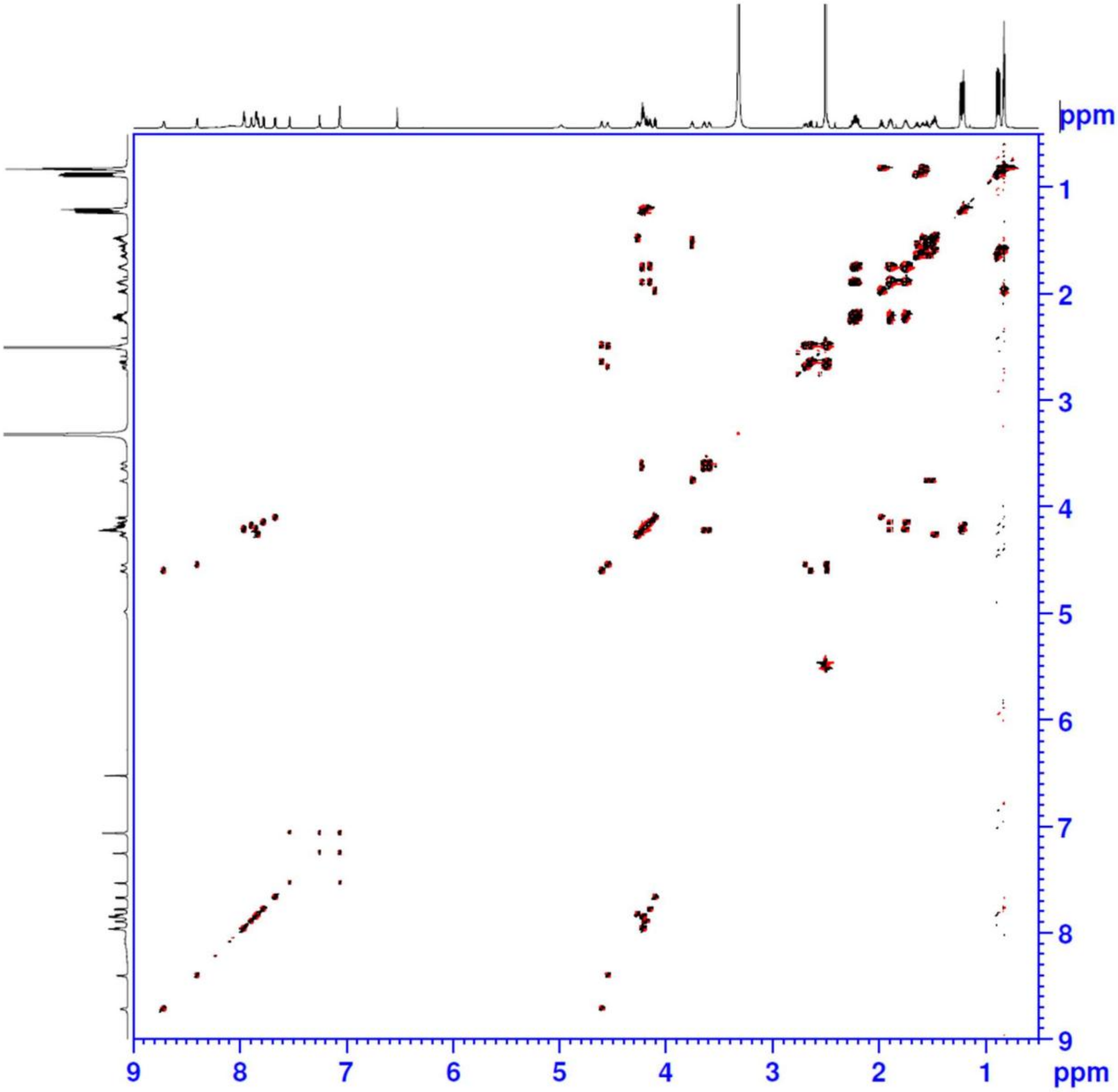

Figure S40. 2D ^{1}H-^{1}H TOCSY spectrum of product **2** (800 MHz, DMSO-*d6*).

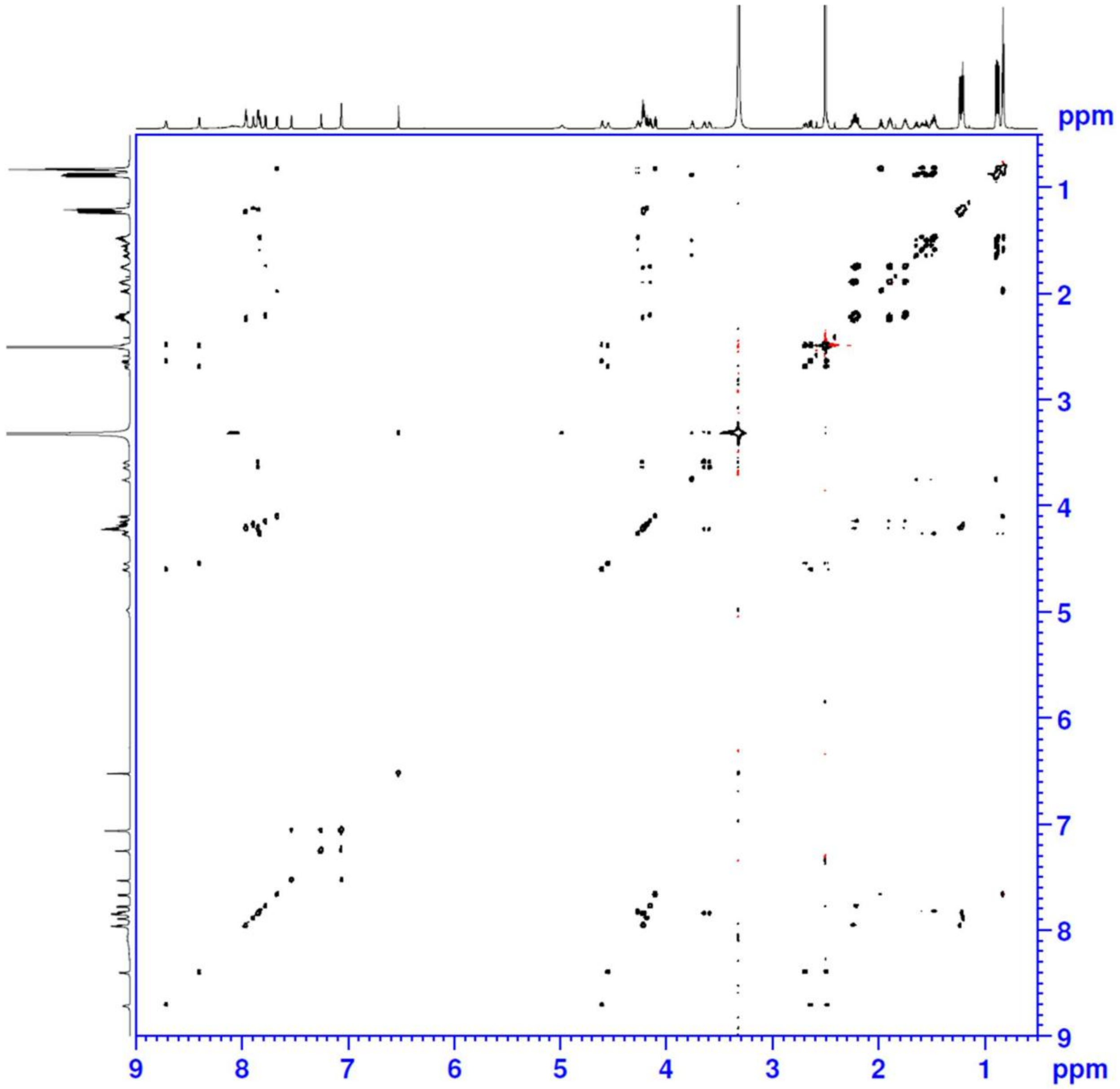

Figure S41. 2D ^{1}H-^{1}H NOESY spectrum of product **2** (800 MHz, DMSO-*d6*).

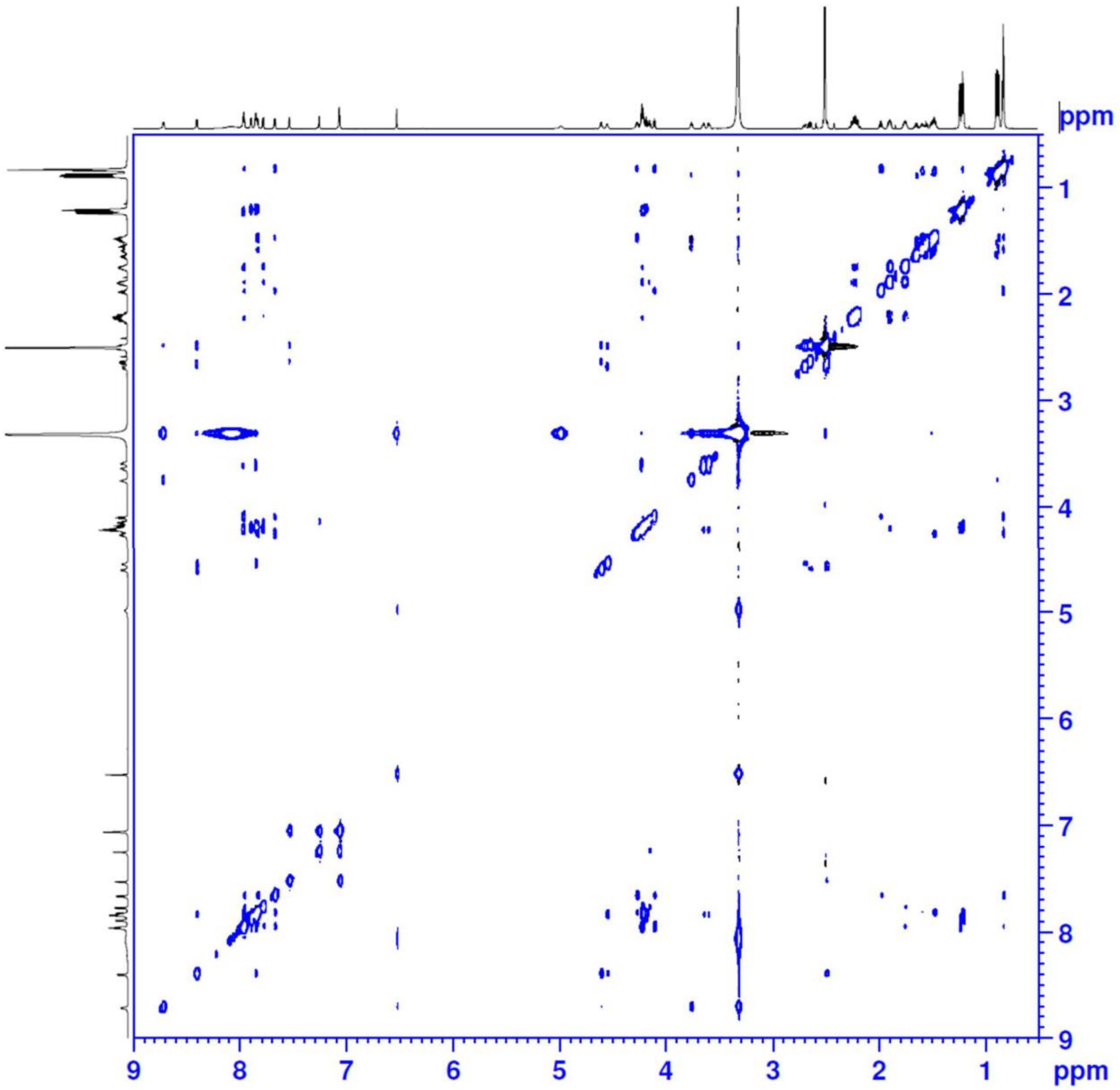

Figure S42. 2D ^{1}H-^{13}C edHSQC spectrum of product **2** (800 MHz, DMSO-*d6*).

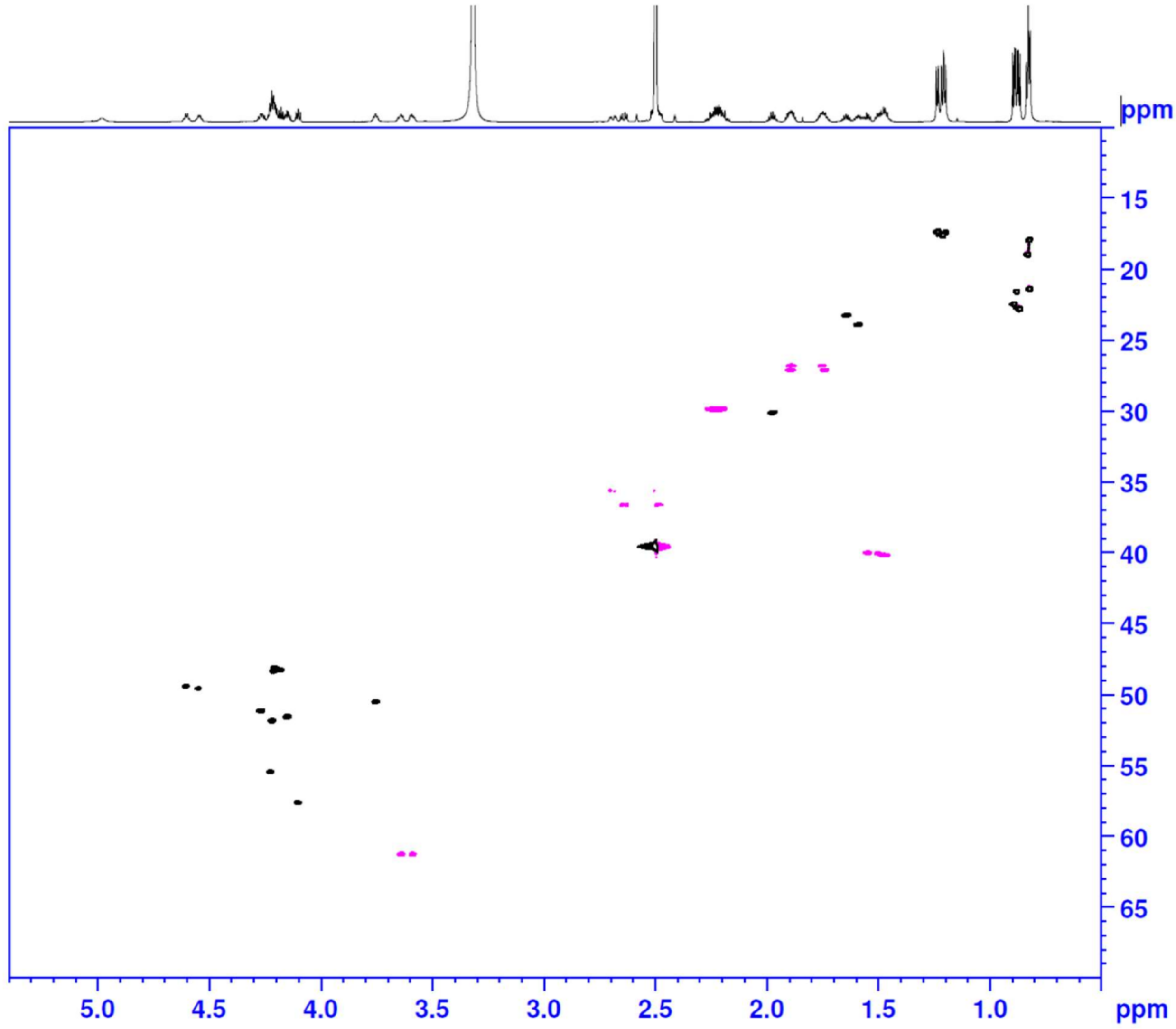

Figure S43. 2D ^{1}H-^{15}N HSQC spectrum of product **2** (800 MHz, DMSO-*d6*).

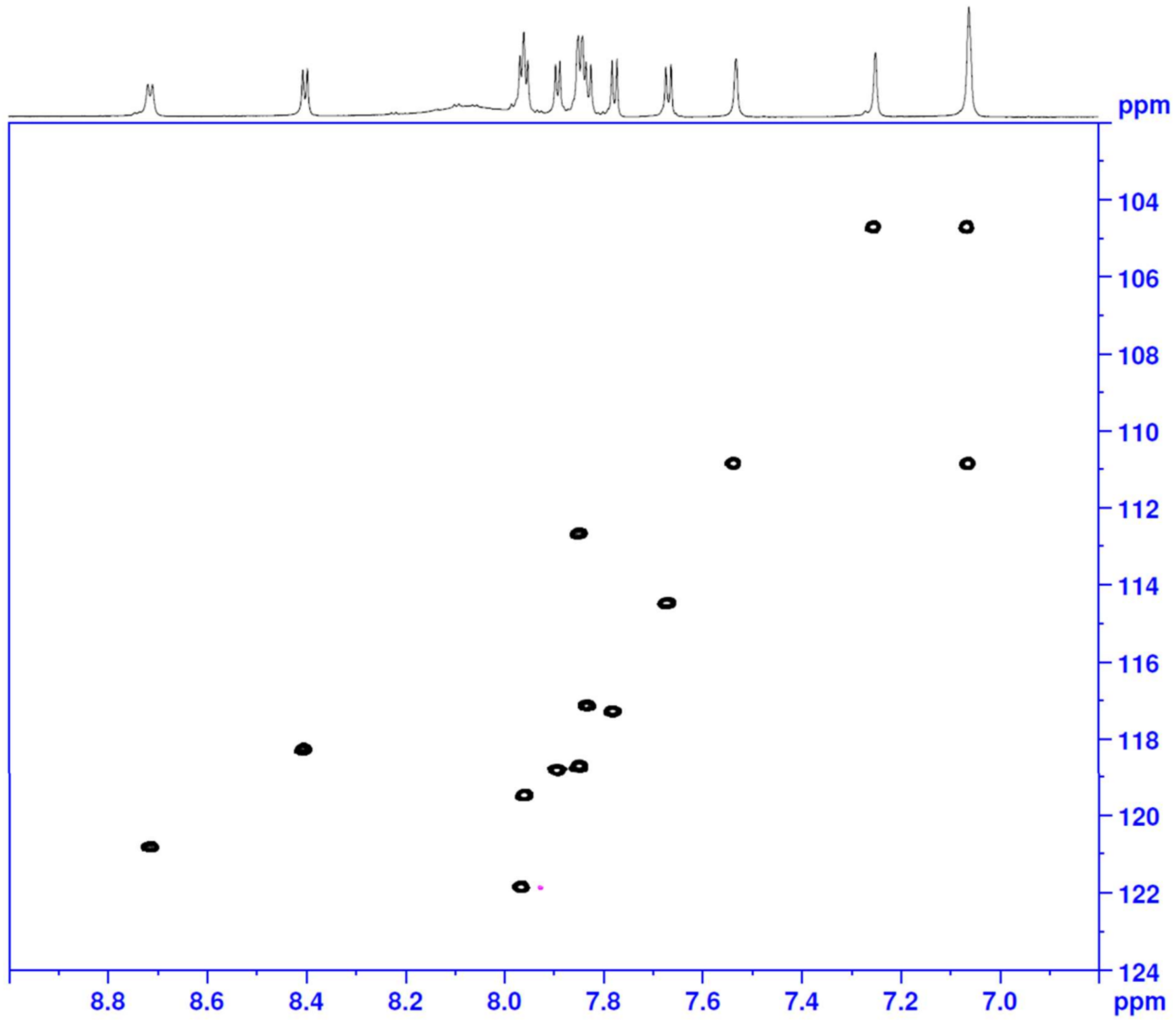

Supplement: Supplementary file 1 [file molecules-31-03097-s001.zip › molecules-4511182-supplementary.pdf]
